# A multiview model for relative and absolute microbial abundances

**DOI:** 10.1111/biom.13503

**Published:** 2021-06-08

**Authors:** Brian D. Williamson, James P. Hughes, Amy D. Willis

**Affiliations:** Department of Biostatistics, University of Washington, Seattle, Washington, USA

**Keywords:** Bayesian estimation, genomics, hierarchical modeling, high throughput sequencing, microbiome

## Abstract

The absolute abundance of bacterial taxa in human host-associated environments plays a critical role in reproductive and gastrointestinal health. However, obtaining the absolute abundance of many bacterial species is typically prohibitively expensive. In contrast, relative abundance data for many species are comparatively cheap and easy to collect (e.g., with universal primers for the 16S rRNA gene). In this paper, we propose a method to jointly model relative abundance data for many taxa and absolute abundance data for a subset of taxa. Our method provides point and interval estimates for the absolute abundance of all taxa. Crucially, our proposal accounts for differences in the efficiency of taxon detection in the relative and absolute abundance data. We show that modeling taxon-specific efficiencies substantially reduces the estimation error for absolute abundance, and controls the coverage of interval estimators. We demonstrate the performance of our proposed method via a simulation study, a study of the effect of HIV acquisition on microbial abundances, and a sensitivity study where we jackknife the taxa with observed absolute abundances.

## 1 | INTRODUCTION

The microorganisms that inhabit a host-associated environment can have a substantial impact on host health (The Human Microbiome Project Consortium, 2012; Libertucci and Young, 2018; Lloyd-Price *et al*., 2019). Each microbial taxon present in an environment has a *bacterial concentration* reflecting the absolute abundance of the taxon per unit volume and the bacterial load on the host. Measuring the concentration of every microbial taxon is resource-intensive: assays must be designed for each taxon and it may not be known *a priori* which taxa are present in an environment. It is therefore common to use assays that can detect many taxa; for example, assays based on a hypervariable region of the 16S rRNA gene or shotgun sequencing of entire microbial communities. While relatively straightforward and inexpensive to perform, these broad range assays do not estimate bacterial concentration. However, concentration is a key quantity of interest in many microbiome studies (Zemanick *et al*., 2010; Stämmler et al., 2016; Vandeputte et al., 2017; Contijoch *et al*., 2019).

While finding the concentration of every microbe in a highly diverse community is challenging, finding the concentration of a small number of microbes may be tractable. For example, bacterium-specific 16S quantitative PCR (qPCR) assays can be developed on a taxon-by-taxon case (see, e.g., Fredricks *et al*., 2007; Ryu *et al*., 2013). When such data are available, the concentration of a small number of microbes could theoretically be combined with relative abundance data to estimate the concentration of all microbial taxa. A method resulting in accurate estimates of all microbial concentrations based on relative abundance data and a small number of microbial concentrations would greatly reduce the labor- and time-intensity of finding the concentration of all microbes in a community. In this paper, we propose and validate a statistical model for this task.

Our approach is to build a hierarchical model that connects the relative abundance data to the absolute abundance data. The observed concentrations of each taxon in each sample are modeled as Poisson-distributed random variables, with taxon- and subject-specific mean parameters that we link to the relative abundances. We observed that 16S sequencing and qPCR assays detected taxa with different efficiencies, and so we incorporate taxon-specific efficiency parameters into our models.

Our paper is structured as follows: the model is defined in Section 2 and estimation is discussed in Section 3. The proposed method is validated on simulated data in Section 4. In Section 5, the proposed estimators are used to model bacterial concentrations in the vaginal microbiome in a HIV acquisition study. We provide concluding remarks in Section 6. Software implementing our model and estimators is available in the R package paramedic (Predicting Absolute and Relative Abundance by Modeling Efficiency to Derive Intervals and Concentrations), available at github.com/statdivlab/paramedic.

## 2 | A MODEL LINKING ABSOLUTE AND RELATIVE ABUNDANCES

Suppose that we have samples from *n* microbial communities. Let the concentration (absolute abundance in, e.g., gene copies per unit volume or colony-forming units per unit volume) of taxon *j* in community *i* be denoted by *μ*_*ij*_, for *i* = 1, …, *n* and *j* = 1, …, *q*. We denote by $\mu \in {\mathbb{R}}_{\geq 0}^{n\times q}$ the matrix of all taxon abundances in all samples. Not all taxa must be present in all communities, and so *μ* may be a sparse matrix.

It is not possible to directly observe *μ* for any taxon because of stochasticity in measuring concentrations (Bonk *et al*., 2018). However, we are able to obtain realizations from a distribution with expectation *μ*_*ij*_. Unfortunately, performing a laboratory experiment to sample taxon concentrations from this distribution for all *j* is not typically possible or is prohibitively expensive. We therefore obtain observed concentrations

(1) $$V_{ij}|\mu_{ij}\sim \text{Poisson}\left(\mu_{ij}\right)$$

for all *i* but only *j* = 1, …, *q*^obs^, where *q*^obs^ < *q*. It is important to distinguish between the true concentration *μ*_*ij*_ and the observed concentration *V*_*ij*_. Even if *μ* > 0, we may observe a zero concentration in any given sample. Stated differently, a zero observed concentration does not imply that the taxon has zero abundance in the community from which the sample was drawn. Note that if covariate data are available, it is straightforward to model *μ*_*ij*_ as a function of these covariates. We illustrate this with an example in Section 5.

While we are not able to observe concentration data for taxa *j* = *q*^obs^ + 1, …, *q*, we are able to collect relative abundance data for all taxa *j* = 1, …, *q*. Let *W*_*ij*_ be the number of sequencing reads (counts) observed from taxon *j* in sample *i*, and *M*_*i*_ = Σ_*j*_ *W*_*ij*_ be the total reads observed from sample *i*. A natural model to connect *W*_*i*·_ ≔ (*W*_*i*1_, …, *W*_*iq*_) to *μ*_*i*·_ ≔ (*μ*_*i*1_, …, *μ*_*iq*_) is

(2) $$W_{i.}|M_{i},\mu_{i.}\sim \text{Multinomial}\left(M_{i},\frac{\mu_{i.}}{\sum_{j=1}^{q}\mu_{ij}}\right).$$

A first-order delta method approximation gives us that under models (1) and (2),

$$E\left[\frac{W_{ij}}{\sum_{k=1}^{q^{\text{obs}}}W_{ik}}\right]\approx \frac{\mu_{ij}}{\sum_{k=1}^{q^{\text{obs}}}\mu_{ik}}\approx E\left[\frac{V_{ij}}{\sum_{k=1}^{q^{\text{obs}}}V_{ik}}\right].$$

**FIGURE 1:**
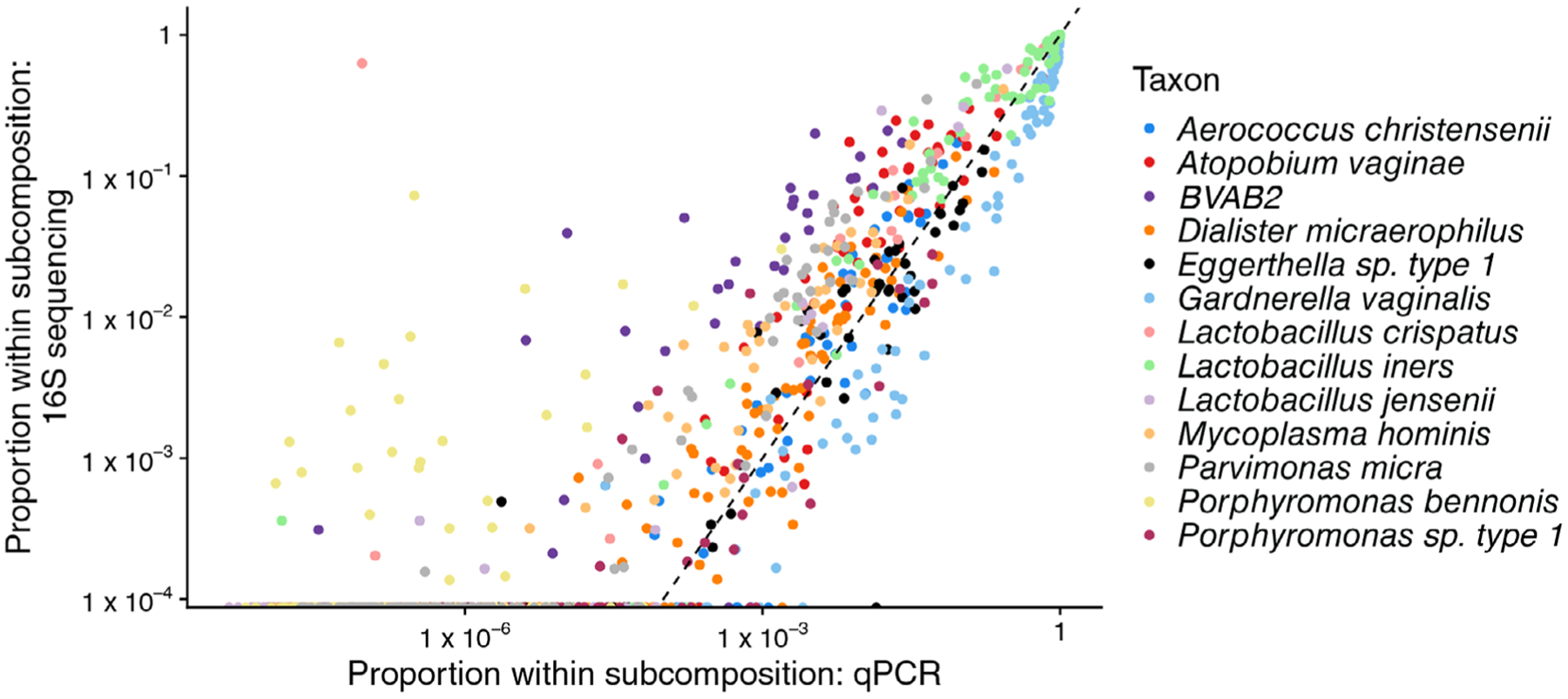
The relative abundance of taxa observed with qPCR versus the relative abundance of the taxa observed by sequencing a hypervariable region of the 16S gene. Note that the subcompositional relative abundance is shown, where the subcomposition is to taxa observed by qPCR. Specifically, $V_{ij}/\sum_{k=1}^{q^{\text{obs}}}V_{ik}$ is plotted against $W_{ij}/\sum_{k=1}^{q^{\text{obs}}}V_{ik}$. In this data set, *q*^obs^ = 13 and *n* = 55.

If this approximation holds, we would expect that a scatterplot of $V_{ij}/\sum_{k=1}^{q^{\text{obs}}}V_{ik}$ versus $W_{ij}/\sum_{k=1}^{q^{\text{obs}}}W_{ik}$ for *i* = 1, …, *n* and *j* = 1, … *q*^obs^ would show random scatter around the *x* = *y* line for each taxon. We show this scatterplot in Figure 1 using data described in Section 5 and do not observe the expected pattern. Instead, we see that $W_{ij}/\sum_{k=1}^{q^{\text{obs}}}W_{ik}$ is proportional to $V_{ij}/\sum_{k=1}^{q^{\text{obs}}}V_{ik}$, but each taxon has a different slope. This suggests that the model (2) is misspecified in expectation, motivating our proposed model

(3) $$W_{i.}|M_{i},\mu_{i.},e\sim \text{Multinomial}\left(M_{i},\frac{e\circ \mu_{i.}}{\sum_{j=1}^{q}e_{j}\mu_{ij}}\right),$$

where ◦ denotes the Hadamard product (pointwise multiplication), *e* ≔ (*e*_1_, …, *e*_*q*_), and *e*_*j*_ is the *efficiency* of taxon *j* for being observed by the relative abundance technology compared to the absolute abundance technology. Our efficiency vector *e* plays the role of the “total protocol bias” parameter of McLaren *et al*. (2019). We now discuss estimation of the parameters of this model, including the identifiability of the efficiencies *e*.

## 3 | ESTIMATING MODEL PARAMETERS

Our primary goal is to construct point and interval estimators for the *μ*_*ij*_ for all *i* and *j*. A secondary goal is to construct prediction interval estimators for the unobserved concentrations *V*_*ij*_ for all *i* and *j* = *q*^obs^ + 1, …, *q*. In this section, we propose three estimation procedures based on the model described in Section 2.

### 3.1 | A simple, efficiency-naïve estimator

A simple estimator of *μ*_*ij*_, the concentration of taxon *j* in sample *i*, is ${\hat{\mu }}_{ij}=s_{i}W_{ij}$, where *s*_*i*_ is a sample-specific scaling factor and we have used the fact that ***E***[*W*_*ij*_] ∝ *μ*_*ij*_. In addition, if *e*_*j*_ for *j* > *q*^obs^ is not estimable, assuming that *e*_*j*_ = *e*_*k*_ for all taxa *j*, *k* may be necessary. An estimate of the scaling factor could then be obtained by considering the implied scaling factor based on aggregating all observed taxa: ${\hat{s}}_{i}=\sum_{j=1}^{q^{\text{obs}}}V_{ij}/\sum_{j=1}^{q^{\text{obs}}}W_{ij}$, yielding the estimator

(4) $${\hat{\mu }}_{ij}^{\text{naïve }}:={\hat{s}}_{i}W_{ij}.$$

While we did not find a reference to estimator (4) in the literature, it is connected to the proposal of Jian *et al*. (2020) (see also Liu *et al*., 2017; Vandeputte et al., 2017; Gibson and Gerber, 2018; Kevorkian *et al*., 2018; Contijoch *et al*., 2019; Morton *et al*., 2019). Jian *et al*. (2020) consider the problem where the total concentration of all bacteria, $\sum_{j=1}^{q}V_{ij}$, is observed for all *i*, and *W*_*ij*_ is also observed for all *i* and *j*. They wish to estimate *μ*_*ij*_ for all *i* and *j*. Their proposed estimator is ${\hat{\mu }}_{ij}=\left(\sum_{j=1}^{q}V_{ij}\right)\times W_{ij}/M_{i}$. Tettamanti Boshier *et al*. (2020) recently validated this proposal using taxon-specific qPCR primers and found it to be “predictive of absolute concentration with certain key exceptions,” such as certain taxa and low biomass (low total bacterial concentration: $\sum_{j=1}^{q}\mu_{ij}$) samples. Bonk *et al*. (2018) give an excellent overview of sources of discrepancies between qPCR and 16S sequencing data.

Previous authors have not proposed methods for quantifying the uncertainty of these naïve estimators. However, interval estimators for *μ*_*ij*_ and prediction interval estimators for ${\left\{V_{ij}\right\}}_{j=q^{\text{obs}}+1}^{q}$ may be constructed by using (1) and (2), the maximum likelihood estimators of the model parameters for *j* ∈ {1, …, *q*^obs^}, and the delta method. We provide a derivation of $\hat{\text{Var}}\left(\text{log}{\hat{\mu }}_{ij}^{\text{naïve }}\right)$ in the Supporting Information (Section SI 1.1). A 100(1 − *α*)% confidence interval for *μ*_*ij*_ may then be constructed as $\text{exp}\left\{\text{log}{\hat{\mu }}_{ij}^{\text{naïve }}\pm q_{1-\alpha /2}\sqrt{\hat{\text{Var}}\left(\text{log}{\hat{\mu }}_{ij}^{\text{naïve }}\right)}\right\}$, where *q*_*γ*_ is the *γ*-quantile of the standard normal distribution. We can additionally form a 100(1 − *α*)% prediction interval for ${\left\{V_{ij}\right\}}_{j=q^{\text{obs}}+1}^{q}$ as $\text{exp}\left\{\text{log}{\hat{\mu }}_{ij}^{\text{naïve }}\pm q_{1-\alpha /2}\sqrt{1/{\hat{\mu }}_{ij}^{\text{naïve }}+\hat{\text{Var}}\left(\text{log}{\hat{\mu }}_{ij}^{\text{naïve }}\right)}\right\}$.

We refer to estimator (4) as the *naïve* estimator because its simplicity must be traded off with its potential drawbacks. First, if the efficiencies are truly unequal, then assuming equal efficiencies will lead to biased estimates of *μ*_*ij*_. It will also lead to invalid interval estimates, because the above intervals were constructed under the assumption of equal efficiencies. Furthermore, these intervals can only be constructed if ${\hat{\mu }}_{ij}^{\text{naïve }}>0$, or equivalently, *W*_*ij*_ > 0. However, 16S data are typically very sparse, with *W*_*ij*_ = 0 for many *i* and *j*, and so the naïve interval estimates cannot be constructed for a large fraction of taxa and samples (in our data set analyzed in Section 5, *W*_*ij*_ = 0 for 77% of the observations). These drawbacks led us to consider more sophisticated estimators, which we now describe.

### 3.2 | A fully Bayesian estimator with variable efficiency

#### 3.2.1 | Point estimation

Bayesian hierarchical modeling is one possible strategy for modeling *V* and *W* to estimate *μ*_*ij*_ and predict *V*_*ij*_ for all *i* and *j*. A hierarchical modeling procedure has several desirable statistical properties here: (i) the joint data model can be customized easily (e.g., to include covariates or to alter the prior distributions); (ii) sampling from the posterior distributions can be performed using freely-available and fast general-purpose software; and (iii) posterior estimates and prediction intervals obtained through this procedure are straightforward to interpret in the context of the generative model. Our goal is to construct valid point and interval estimators in the presence of potentially unequal efficiencies and when *W*_*ij*_ = 0.

To reflect the differing efficiencies with which taxa are detected by 16S and qPCR data (see, e.g., Figure 1) we consider the following model:

(5) $$V_{ij}|\mu_{ij}\sim \text{Poisson}\left(\mu_{ij}\right)\text{ and }W_{i.}|M_{i},\mu_{i.},\;e\sim \text{Multinomial}\left(M_{i},p_{i}\right),\text{ where }\;p_{ij}=\frac{\mu_{ij}e_{j}}{\sum_{\ell =1}^{q}\mu_{i\ell }e_{\ell }}$$

for all *i* and *j*. If covariate data are available, the model can be adapted to model *μ*_*ij*_ as a function of these covariates (e.g., see Section 5). Furthermore, if the samples were obtained in multiple batches, the efficiencies can be modeled as batch-dependent. Examples of how to customize the model are available in the paramedic package documentation.

In the absence of covariate or batch information, we propose the following prior distributions of the parameters *μ*_*ij*_ and *e*_*j*_. Since there is often substantial right skew in the observed *V*_*ij*_ (see Section 5), and to ensure positivity of the concentration *μ*_*ij*_, we propose a hierarchical lognormal prior on the *μ*_*i*·_ with hyperparameters *β* and Σ (a diagonal matrix): log *μ*_*i*·_ ~ *N*_*q*_(*β*, Σ), where $\beta \sim N_{q}\left(0,\sigma_{\beta }^{2}\right)$ and $\Sigma_{jj}\sim \text{Lognormal}\left(0,\sigma_{\Sigma }^{2}\right)$. We model $e_{j}\sim \text{Lognormal}\left(0,\sigma_{e}^{2}\right)$, where $\sigma_{e}^{2}\sim \text{InverseGamma}\left(\alpha_{\sigma },\kappa_{\sigma }\right)$. This soft-centering approach makes the parameters *e*_*j*_ and *μ*_*i*·_ identifiable. We note that samples from the posterior distribution of *e*_*j*_ need not satisfy the property that $\sum_{j=1}^{q^{\text{obs}}}\text{log}e_{j}=0$ nor that $\sum_{j=1}^{q}\text{log}e_{j}=0$ exactly, though we find that both summations are close to zero in practice. We also investigated a hard-centering approach using the model $e_{j}\sim \text{Lognormal}\left(0,\sigma_{e}^{2}\right)$, $\sigma_{e}^{2}\sim \text{InverseGamma}\left(\alpha_{\sigma },\kappa_{\sigma }\right)$, and $e_{j}={\tilde{e}}_{j}/\text{exp}\left(\frac{1}{q^{\text{obs }}}\sum_{j\prime =1}^{q^{\text{obs}}}\text{log}{\tilde{e}}_{j\prime }\right)$. However, we found little difference between the point and interval estimates obtained from the hard- and soft-centering approaches, and similarly for hard-centering over all taxa $\left(e_{j}={\tilde{e}}_{j}/\text{exp}\left(\frac{1}{q}\sum_{j\prime =1}^{q}\text{log}{\tilde{e}}_{j\prime }\right)\right)$. Throughout this manuscript we show results for the soft-centering approach. An empirical comparison with the hard-centering approach can be found in the Supporting Information (Section SI 3.1).

We discuss our default choices of $\sigma_{\beta }^{2}$, $\sigma_{\Sigma }^{2}$, *α*_*σ*_, and *κ*_*σ*_ in Section 4. In practice, these hyperparameters may be based on independently observed data, numerical experiments, expert opinion, or a combination of these three. See the Supporting Information (Section SI 3.3) for an investigation of the sensitivity of results to the chosen hyperparameters.

We fit hierarchical model (5) using Stan (Carpenter *et al*., 2017). Stan is an imperative probabilistic programming language that uses assignment and sampling statements to specify a log-density function. Fully Bayesian inference is available using Hamiltonian Monte Carlo sampling; point estimates may additionally be computed using optimization. Since our parameter space (*μ*, *β*, Σ_11_, …, Σ_*qq*_, $\sigma_{e}^{2}$) is continuous and the model described above may need to be customized based on the data-generating mechanism, Stan is ideal for fitting our model. After fitting the model, we obtain samples from the joint posterior distribution.

#### 3.2.2 | Interval construction

We now discuss obtaining interval estimates for *μ*_*ij*_ and prediction interval estimates for *V*_*ij*_ using the fitted model. Let 1 − *α* denote the desired level for intervals.

Credible intervals for *μ*_*ij*_ are constructed via the (*α*/2, 1 − *α*/2)-quantiles of the posterior sampling distribution of *μ*_*ij*_ based on our proposed hierarchical model.

Prediction intervals can be computed in two ways. We incorporate the hierarchical uncertainty of our proposed model into a Wald-type interval estimate based on ${\hat{V}}_{ij}$. Using the law of iterated variance conditional on the true *μ*_*ij*_ and our model that *V*_*ij*_ ~ *Poisson*(*μ*_*ij*_), we estimate the variance in the prediction ${\hat{V}}_{ij}$ as $\hat{\text{Var}}\left({\hat{V}}_{ij}\right):={\hat{\mu }}_{ij}+\hat{\text{Var}}\left(\mu_{ij}\right)$, where $\hat{\text{Var}}\left(\mu_{ij}\right)$ is the variance of the posterior sampling distribution of *μ*_*ij*_ and ${\hat{\mu }}_{ij}$ is the posterior mean. Then our prediction intervals for *V*_*ij*_ are $\text{max}\left(0,{\hat{V}}_{ij}\pm \Phi^{-1}\left(1-\frac{\alpha }{2}\right)\sqrt{\hat{\text{Var}}\left({\hat{V}}_{ij}\right)}\right)$, where Φ^−1^(*γ*) is the *γ*-quantile of the standard normal distribution. We truncate the lower limit of the prediction interval at zero to reflect that bacterial concentrations are nonnegative. We also investigated a quantile-based approach for prediction interval construction, but found its performance to be extremely similar to the Wald-type prediction intervals. We outline the quantile-based approach in the Supporting Information (Section SI 1.2).

#### 3.2.3 | An efficiency-naïve estimator

A simplified model may easily be obtained by assuming that all of the efficiencies are equal:

(6) $$V_{ij}|\mu_{ij}\sim \text{Poisson}\left(\mu_{ij}\right)\text{ and }W_{i.}|M_{i},\mu_{i.},\;e\sim \text{Multinomial}\left(M_{i},p_{i}\right),\text{ where }\;p_{ij}=\frac{\mu_{ij}}{\sum_{\ell =1}^{q}\mu_{i\ell }}$$

for all *i* and *j*. We use this model in simulated examples for simplicity in cases with equal efficiencies and to highlight the negative consequences of assuming equal efficiencies when efficiencies are truly unequal. We suggest that model (5) always be used.

#### 3.2.4 | Advantages of the varying-efficiency model

Before comparing and validating each of these models and estimators on simulated and observed data, we briefly note some of the advantages of our proposed varying-efficiency model compared to existing and naïve approaches. First, we connect the relative abundance and absolute abundance via a statistical model. Second, by modeling the efficiencies explicitly, we account for the fact that the relative abundances are proportional to the absolute abundances but with a taxon-specific slope, as we observed in Figure 1. Our proposal naturally incorporates the additional uncertainty associated with the unknown efficiencies into our interval estimators. Our relative abundance parameters obey the constraint that $\sum_{j=1}^{q}p_{ij}=1$ for all *i*. Finally, by adopting a Bayesian hierarchical modeling approach, we can obtain the posterior distribution of *μ*_*ij*_, *j* = *q*^obs^ + 1, …, *q*. In other words, we are able to estimate the concentration of taxa for which we do not have absolute abundance data, and construct interval estimators for the concentration of those taxa even when the observed relative abundance is zero. The posterior distribution of the concentration of taxon *j* for *j* > *q*^obs^ will be driven by *W*_*ij*_ and *V*_*ij*_ for *j* ≤ *q*^obs^, and the prior parameters $\sigma_{\beta }^{2}$, $\sigma_{\sum }^{2}$, *α*_*σ*_, and *κ*_*σ*_. We note that the interval estimates for *μ*_*ij*_ and *V*_*ij*_ can be wide for *j* > *q*^obs^.

## 4 | RESULTS UNDER SIMULATION

We now present simulation results on the performance of the estimators proposed in Section 3. In all cases, we use Stan to fit hierarchical models (5) and (6) using four chains per simulated data set, each with 10,000 burn-in iterations and 10,500 total iterations (2000 total iterations for each of *B* = 50 simulations for each set of parameters to investigate). We describe our process for initializing these chains in the Supporting Information (Section SI 2). We ran our simulation study on a high-performance computing cluster of Linux nodes each with at least four cores and 16 GB of memory (each individual simulation replicate may have been allocated less memory at run-time). Each iteration ran for between approximately 0.4 and 3 s, with variation due to both memory allocation and data structures. It was not feasible to confirm convergence for every individual simulation via trace plots, and so we confirmed that the median and interquartile range (IQR) of the Gelman–Rubin $\hat{R}$ statistic (Gelman and Rubin, 1992) was close to 1 for all parameters of interest.

We assess performance for each Monte Carlo replicate using root mean squared error (RMSE) for *μ*_*ij*_ and empirical coverage of nominal 95% credible intervals for *μ*_*ij*_, both averaged over all *n* samples and *q* taxa; and root mean squared prediction error (RMSPE) for *V*_*ij*_ and empirical coverage of nominal 95% prediction intervals for *V*_*ij*_, both for *j* = *q*^obs^ + 1, …, *q* and averaged over both *n* and *q*. The exact specification of these performance measures is provided in the Supporting Information (Section SI 3). While our primary goal is estimation of the true concentration *μ*, we also investigate the performance of predicting *V* for the unobserved taxa, as this may be of interest in some settings (e.g., for assessing correct model specification; see Section 5.3 and Figure 6).

We report these four summaries for each estimator under consideration. In each case, we display the average of the summary measure over Monte Carlo replicates. In all simulations, we exclude taxa whose mean expected abundance *μ*_*ij*_, averaged over all samples, is below 1 unit. In practice, taxa observed in low abundance across all samples are typically excluded from analysis (Callahan *et al*., 2016), and so this reflects the typical use case of the proposed method. However, in practice *μ*_*ij*_ is unknown, and thus exclusion may be done based on *W*_*ij*_. We provide a discussion of filtering rules and the rationale behind the particular rule used here in the Supporting Information (Section SI 3.2). Finally, if the naïve estimate for a given sample and taxon is zero, then we do not include that sample-taxon pair when computing average coverage of naïve interval estimates.

### Default parameters:

We strongly recommend that the user investigate the sensitivity of results to prior parameters. In addition, the values of prior parameters should be carefully chosen to match the measurement scale of the data set. In our data set of Section 5, the sample variances of the realized log-qPCR data are near 50. Based on this observation, we chose $\sigma_{\beta }^{2}=50$ and $\sigma_{\sum }^{2}=50$ as default parameters for our simulation study. We additionally chose *α*_*σ*_ = 2 and *κ*_*σ*_ = 1 since these choices led to fast convergence of our sampling algorithm in our simulated data sets. We provide an investigation of sensitivity to the prior parameters ($\sigma_{\beta }^{2}$, $\sigma_{\sum }^{2}$) and (*α*_*σ*_, *κ*_*σ*_) in the Supporting Information (Section SI 3.3).

### Simulation settings:

Throughout this section, we simulate data according to *M*_*i*_ ~ DiscreteUniform(10^4^, 10^5^), reflecting the distribution of read depths that we observed in our data. We also simulate data according to $\text{log}\mu_{i.}\overset{\;iid\;}{\sim }N_{q}(\beta ,\Sigma )$ for all subjects *i* = 1, …, *n* where $\beta_{j}\overset{\;iid\;}{\sim }N\left(0,\sigma^{2}=50\right)$ for all *j* and Σ = **I**_*q*_. In all cases, we simulate *V*_*ij*_ ~ *Poisson*(*μ*_*ij*_) and *W*_*i*·_ ~ *Multinomial* (*M*_*i*_, *p*_*i*·_), where $p_{ij}=\frac{\mu_{ij}e_{j}}{\sum_{j=1}^{q}\mu_{ij}e_{j}}$. The specific choices for the distribution of *e*_*j*_ and the values of *q* and *q*^obs^ vary in each simulation. We used R version 3.4.3 in all analyses in this paper.

### 4.1 | Effect of varying the number of taxa

We first investigate the effect of varying *q* and *q*^obs^ while holding other parameters fixed. We simulated data with no varying efficiency (*e*_*j*_ = 1 for all *j*) and fit the efficiency-naïve model (6) for simplicity. We investigate the varying-efficiency model in Section 4.2.

We observe ${\left\{V_{ij}\right\}}_{j=1}^{q^{\text{obs }}}$ and ${\left\{W_{ij}\right\}}_{j=1}^{q}$ for *i* = 1, …, *n*, where *n* = 100. We vary *q* ∈ {10, 20, 40, 60}; for each *q*, we additionally vary *q*^obs^ ∈ {2, 3, …, 7}. For each unique combination of *q* and *q*^obs^, we generate data from this population by: (i) generating *β* and Σ; and (ii) generating independent Monte Carlo replicates of *μ*_*ij*_, *V*_*ij*_, *M*_*i*_, and *W*_*ij*_.

qPCR data are typically available for only the taxa that are of most interest to the investigator or are expected to be most abundant. For this reason, in our simulations the *q*^obs^ most abundant taxa based on the observed *W*_*ij*_, averaged over the *n* samples, are used to estimate *μ* for all taxa and predict the unobserved qPCR data, ${\left\{V_{ij}\right\}}_{j=q^{\text{obs }}+1}^{q}$. This means that in our simulations, as *q* increases we add increasingly rare taxa.

**FIGURE 2:**
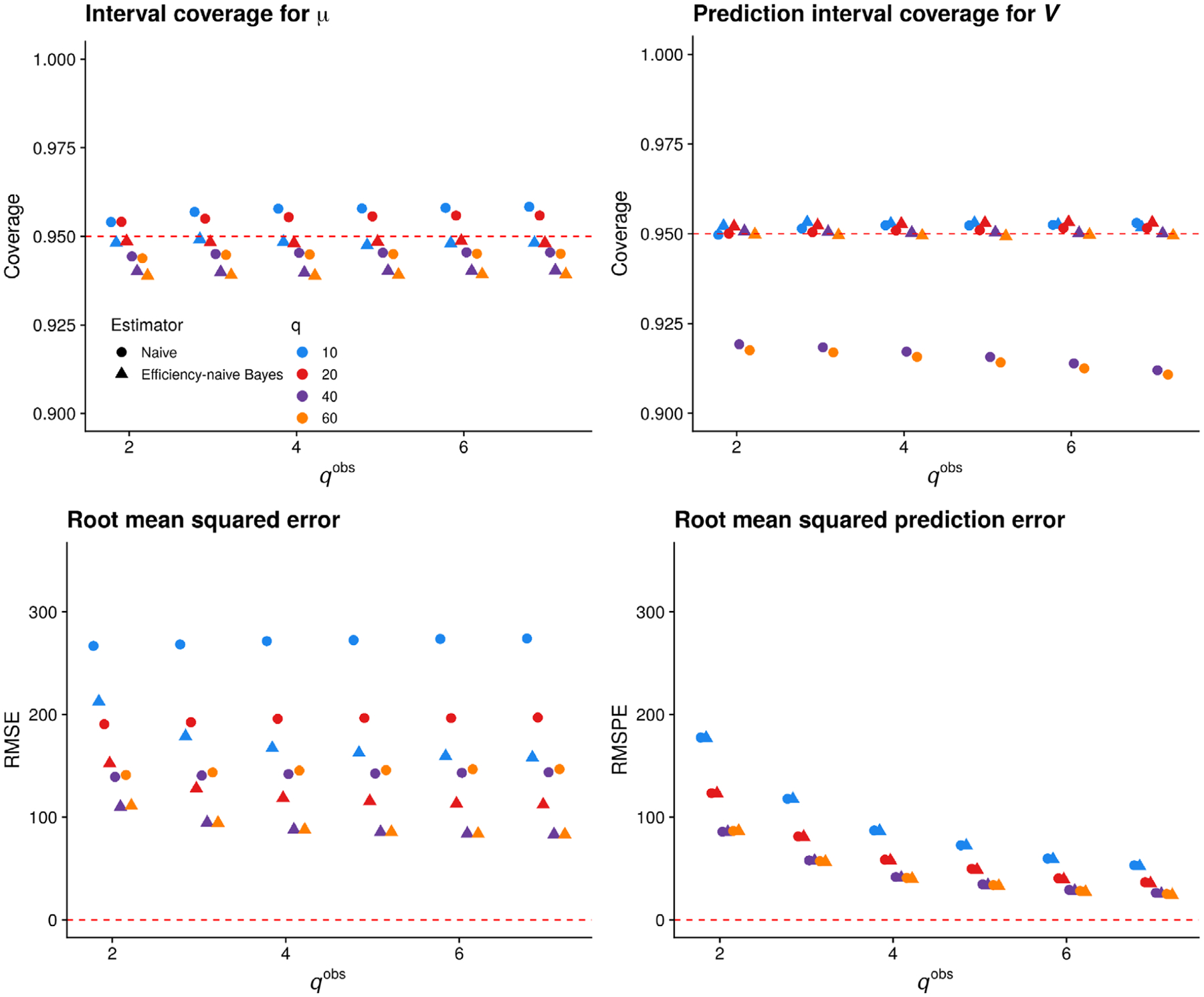
Performance of the naïve estimator (circles) and proposed efficiency-naïve Bayesian estimator (triangles) versus *q*^obs^ for *q* ∈ {10, 20, 40, 60}. Top row: coverage of nominal 95% intervals based on both estimators. Bottom row: root mean squared error and root mean squared prediction error for both estimators. In each plot, color denotes *q*, while shape denotes the estimator.

Figure 2 displays the results of this experiment. In the top row, we see that nominal 95% intervals for *μ* based on the naïve estimator have slightly greater average coverage than credible intervals based on the proposed efficiency-naïve Bayesian estimator. However, the average coverage of the efficiency-naïve credible intervals for *μ* is close to nominal for all (*q*, *q*^obs^) combinations. We note that for both estimators, average coverage for *μ* decreases as *q* increases for a fixed *q*^obs^. This is due to poor marginal coverage for the lowest abundance taxa (see Supporting Information, Section SI 3.4). We also see that average coverage of prediction intervals for *V* based on the proposed efficiency-naïve estimator is at the nominal level for all (*q*, *q*^obs^) combinations. This is encouraging, especially in view of the fact that we often have many more relative abundance measurements than species-specific qPCR measurements; indeed, the results we present in Section 5 are based on *q*^obs^ = 13. In contrast, average coverage of prediction intervals based on the naïve estimator is below the nominal level for large *q*; this is due in large part to the fact that a naïve interval does not exist when the naïve estimator equals zero. The proportion of cases where the naïve estimator is zero, and thus excluded from computing performance, is 0.17%, 1.5%, 26%, and 50% of sample-taxon pairs for *q* = 10, 20, 40, and 60, respectively. In addition, since we compute intervals based on the naïve estimator on the log scale, the lower limit of the backtransformed interval is almost surely greater than zero, if the interval exists. This leads to undercoverage of cases where the true qPCR value is exactly zero, which is increasingly the case as *q* increases. In the bottom row of Figure 2, we see that the efficiency-naïve estimator has lower RMSE than the naïve estimator over all (*q*, *q*^obs^) combinations, while the RMSPE of the two estimators is comparable. As *q*^obs^ increases for a fixed *q*, both RMSE and RMSPE tend to decrease. We provide evidence in Section SI 3.5 that the efficiency-naïve estimator has low bias, and thus the RMSE of this estimator appears to be driven by its variance.

After averaging over Monte Carlo replicates, the median Gelman–Rubin $\hat{R}$ for *μ* over all samples and taxa for *q* = 60 and *q*^obs^ = 7 was 0.99, with an IQR of [0.99, 1.00], showing excellent convergence; convergence was similar in other pairings of *q* and *q*^obs^ and for *β* and Σ for each pairing. We investigated the trace plots for a small number of Monte Carlo samples, which showed well-mixed chains after the burn-in period.

In many experiments, *q* may be much larger than 60. For example, in our data analysis of Section 5, *q* = 127. We anticipate that the trends observed in this simulated experiment would hold for larger *q*, but did not investigate them here because the time required to compute our estimator increases with *q*.

### 4.2 | Varying the distribution of efficiency

In this experiment, we fix *q* = 40 and *q*^obs^ = 7. We vary *σ*_*e*_ ∈ {0, 0.1, …, 0.5, 0.6, 0.8, 1}. For each *σ*_*e*_, we generate data from this population in the same manner as the previous experiment, resulting in 50 independent Monte Carlo replicates. We use Stan to fit our proposed variable-efficiency model (5) and our efficiency-naïve model (6). As we have described before, the naïve estimator does not account for varying efficiency.

**FIGURE 3:**
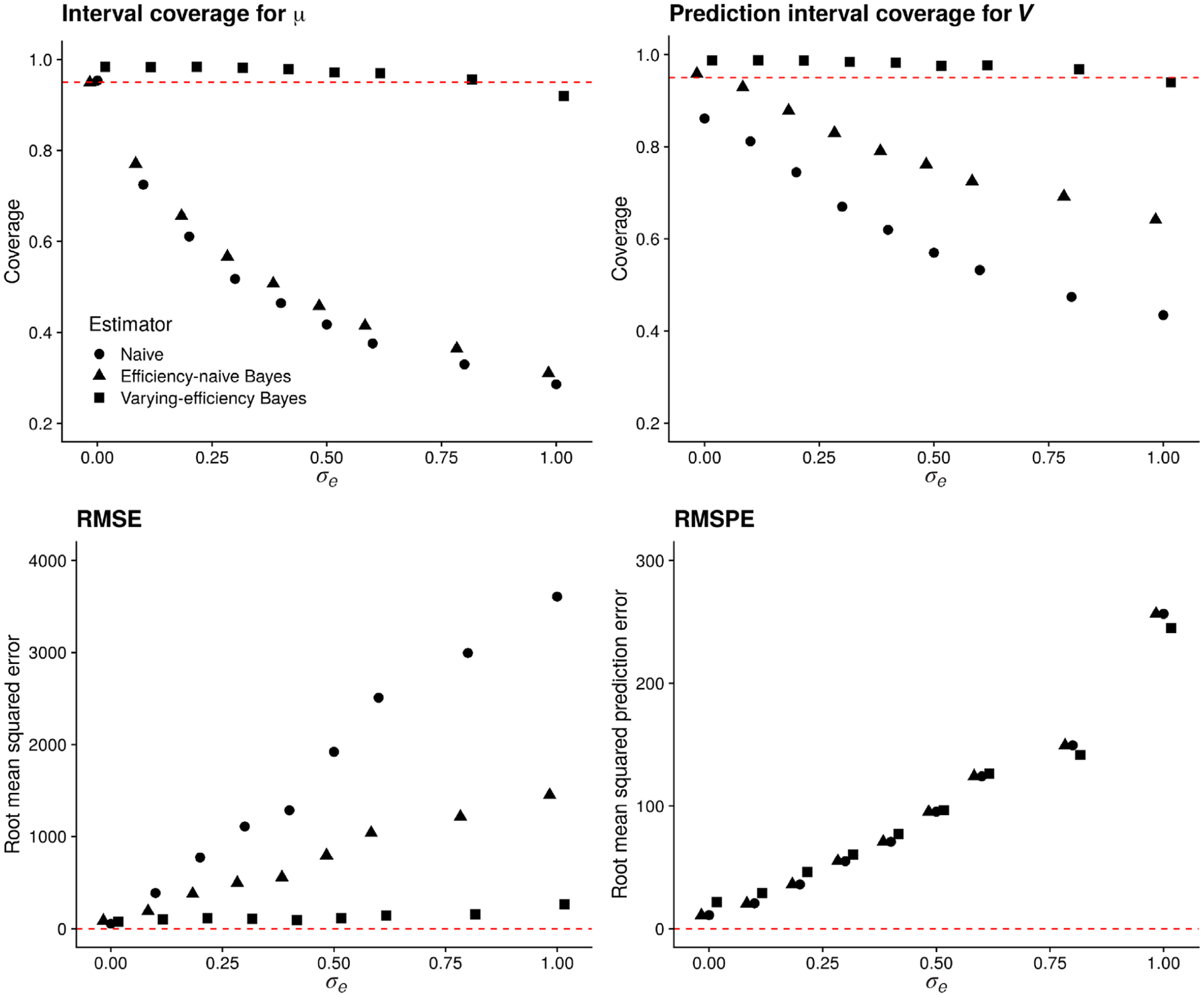
Performance of the naïve estimator (circles), efficiency-naïve Bayesian estimator (triangles), and varying-efficiency Bayesian estimator (squares) versus *σ*_*e*_ for *q* = 40 and *q*^obs^ = 7. Top row: coverage of nominal 95% intervals based on each estimator. Bottom row: root mean squared error and root mean squared prediction error for all estimators

Figure 3 displays the results of this experiment. In the top row, we see that as *σ*_*e*_ increases, the prediction interval average coverage and credible interval average coverage decline to levels below 95% for the naïve and efficiency-naïve Bayesian models but are maintained close to or above 95% for the proposed varying-efficiency Bayesian model. This coincides with our expectation that varying efficiency must be modeled if it is truly present. In the bottom row, we see that as *σ*_*e*_ increases, the RMSE and RMSPE of all three estimators increases. The varying-efficiency Bayesian estimator tends to have the lowest RMSE. While the RMSPE of the varying-efficiency estimator is highest at small values of *σ*_*e*_, at moderate and high levels of varying efficiency (*σ*_*e*_ > 0.5) the RMSPE of this estimator is comparable to or below that of the efficiency-naïve Bayesian and naïve estimators. Since we observed nearly identical patterns for the same experiment with *q*^obs^ = 3, we do not show those results here. In the data we analyze in Section 5, we estimate ${\hat{\sigma }}_{e}=1.74$. This suggests that interval estimates based on the proposed varying-efficiency Bayesian estimator will be more reliable with respect to interval coverage on this data set.

After averaging over Monte Carlo replicates, the median Gelman–Rubin $\hat{R}$ for *μ* over all samples and taxa for *σ*_*e*_ = 0.5 was 1.00 (IQR [0.99, 1.00]) when varying efficiency was modeled and 0.99 (IQR [0.99, 1.00]) when efficiency was not modeled. As we varied *σ*_*e*_, the median $\hat{R}$ for all model parameters tended to be near one, with a maximum of 1.2 for *β* when *σ*_*e*_ = 0 and varying efficiency was not modeled. Inspection of trace plots for a small number of samples showed well-mixed chains after the burn-in period.

In the Supporting Information (Section SI 3.3), we investigate the effect of the efficiency hyperparameters *α*_*σ*_ and *κ*_*σ*_ on coverage and interval width for *V*, *μ*, and *e*. In brief, we found that overconcentrating priors on efficiency reduces interval width at the expense of coverage.

### 4.3 | Additional empirical results

We also investigated the performance of our proposed procedure under model misspecification in the Supporting Information (Section SI 3.6). The coverage of our method is relatively robust to misspecifying the distribution of *e*, somewhat robust to mild misspecification of the distribution of *μ*, but not robust to significant departures from the distribution of *μ*.

## 5 | RESULTS FROM A STUDY OF THE VAGINAL MICROBIOME

### 5.1 | Description of the study sample

These data are from a case-control study of 110 study participants from eastern and southern Africa, described in McClelland *et al*. (2018). Cases are defined as women who acquired HIV during the study, while controls are defined as women without HIV infection.

The data contain observed concentrations from qPCR (measured in 16S gene copies per swab) on *q*^obs^ = 13 taxa: *Aerococcus christensenii*, *Atopobium vaginae*, *BVAB2 spp*., *Dialister micraerophilus*, *Eggerthella spp. type 1*, *Gardnerella vaginalis*, *Lactobacillus crispatus*, *Lactobacillus iners*, *Lactobacillus jensenii*, *Mycoplasma hominis*, *Porphyromonas spp. type 1*, *Porphyromonas bennonis*, and *Parvimonas micra*. The 16S sample processing protocols are described in McClelland *et al*. (2018), and *q* = 127 after 5% prevalence filtering (Callahan *et al*., 2016). To reflect limits on computation time and computing memory (see Section SI 4 for details and Section 6 for a discussion), we uniformly-at-random selected *n* = 55 samples to analyze using our proposed method. The goals of this analysis were to: (i) estimate the true concentrations *μ* for all 127 taxa and each of the 55 samples; (ii) predict the bacterial concentrations for taxa *j* > *q*^obs^ in each sample, and (iii) estimate the expected change in the log concentration of each taxon for samples from HIV cases compared to control cases.

We fit the model log *μ*_*i*·_ ~ *N*_*q*_(*β*_0_ + *β*_1_*X*_*i*_, Σ), for *i* = 1, …, *n*, where $\beta_{0}\in {\mathbb{R}}^{q}$, $\beta_{1}\in {\mathbb{R}}^{q}$, and *X*_*i*_ = 1 if subject *i* is HIV-positive and *X*_*i*_ = 0 otherwise. We chose prior distributions $\beta_{0}\sim N_{q}\left(0_{q},\sigma_{\beta_{0}}^{2}I_{q}\right)$, and $\beta_{1}\sim N_{q}\left(0_{q},\sigma_{\beta_{1}}^{2}I_{q}\right)$, where **0**_*q*_ is a *q*-dimensional column vector containing all zeros and **I**_*q*_ is the *q* × *q* identity matrix. We use the prior distribution for Σ described in Section 3.2.1. The ease of fitting this covariate-adjusted model highlights an advantage of using Stan to estimate the model parameters. We fit our model using four chains, each with 18,000 burn-in iterations and 20,000 total iterations. We selected hyperparameters $\sigma_{\beta_{0}}=1.62$, $\sigma_{\beta_{1}}=1$, and $\sigma_{\Sigma }=\sqrt{50}$ based on the observed data; we additionally selected *α*_*σ*_ = 4 and *κ*_*σ*_ = 3. A sensitivity analysis to the chosen hyperparameters can be found in the Supporting Information (Section SI 5). In addition to fitting this covariate-adjusted model, we also fit the unadjusted model from Section 3.2.1 and found that the estimated *μ*_*ij*_’s are extremely similar across the two methods, with a mean difference of 5.8%. However, the widths of the interval estimates for *μ*_*ij*_ from the unadjusted model are on average approximately 6.4% wider than those from the covariate-adjusted model. Details on this analysis are given in the Supporting Information (Section SI 5). We ran our data analyses on a high-performance computing cluster of Linux nodes each with at least six cores and 60 GB of memory, and each iteration took approximately 1.3 min to complete.

### 5.2 | Results of the primary analysis

**FIGURE 4:**
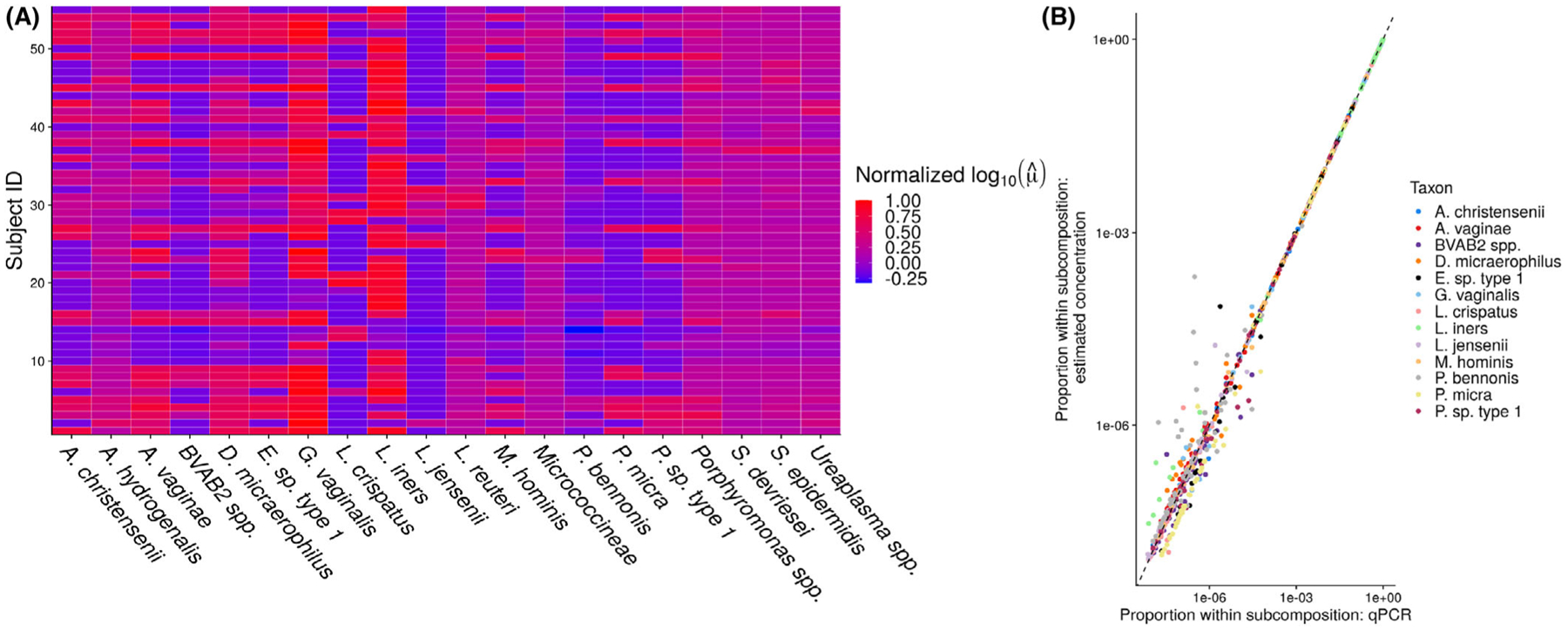
(A) A heatmap showing posterior mean log concentrations for 20 taxa (the 13 taxa with observed qPCR and seven randomly sampled taxa) and all 55 samples. Red indicates large concentration relative to the maximum in this subsample, while blue indicates small concentration relative to the maximum in this subsample. (B) The relative abundance of taxa observed with qPCR versus the estimated relative abundance of the taxa based on the variable-efficiency estimator. Specifically, $V_{ij}/\sum_{k=1}^{q^{\text{obs}}}V_{ik}$ is plotted against ${\hat{\mu }}_{ij}/\sum_{k=1}^{q^{\text{obs}}}{\hat{\mu }}_{ik}\cdot q^{\text{obs}}=13$ and *n* = 55 in this data set.

Figure 4 displays the results of our primary analysis. Panel A (left) shows the posterior means of the log concentrations for 20 taxa (the 13 taxa with observed qPCR data plus seven randomly-sampled taxa) and all 55 samples. Red denotes large normalized log concentration, while blue denotes small normalized log concentration. This figure appears in color in the electronic version of this paper, and any mention of color refers to that version. We observe substantial variability in concentrations both between samples and between taxa. For example, while *L. iners* appears to be a high-abundance taxon on average, some samples (e.g., samples 2 and 4) have much smaller concentration. This pattern appears more striking in the taxa lacking qPCR measurements: for example, some samples have a large estimated abundance of *Porphyromonas spp*. (e.g., samples 3 and 36), while many others have a low estimated abundance of this same taxon. Interval estimates for *μ*_*ij*_ and prediction intervals for *V*_*ij*_ are available as Supplementary Data. Panel B (Figure 4, right) plots $V_{ij}/\sum_{k=1}^{q^{\text{obs}}}V_{ik}$ against ${\hat{\mu }}_{ij}/\sum_{k=1}^{q^{\text{obs}}}{\hat{\mu }}_{ij}$. We see that the model produces reasonable estimates of *μ* on the taxa for which we have qPCR data. We estimate that ${\hat{\sigma }}_{e}=1.74$, with a 95% credible interval of (1.00, 2.87). We estimate that the efficiencies of the taxa with observed qPCR data range between 0.16 and 39.86. These results together imply that there is substantial variation in taxon efficiencies, and that modeling this variation is important.

**FIGURE 5:**
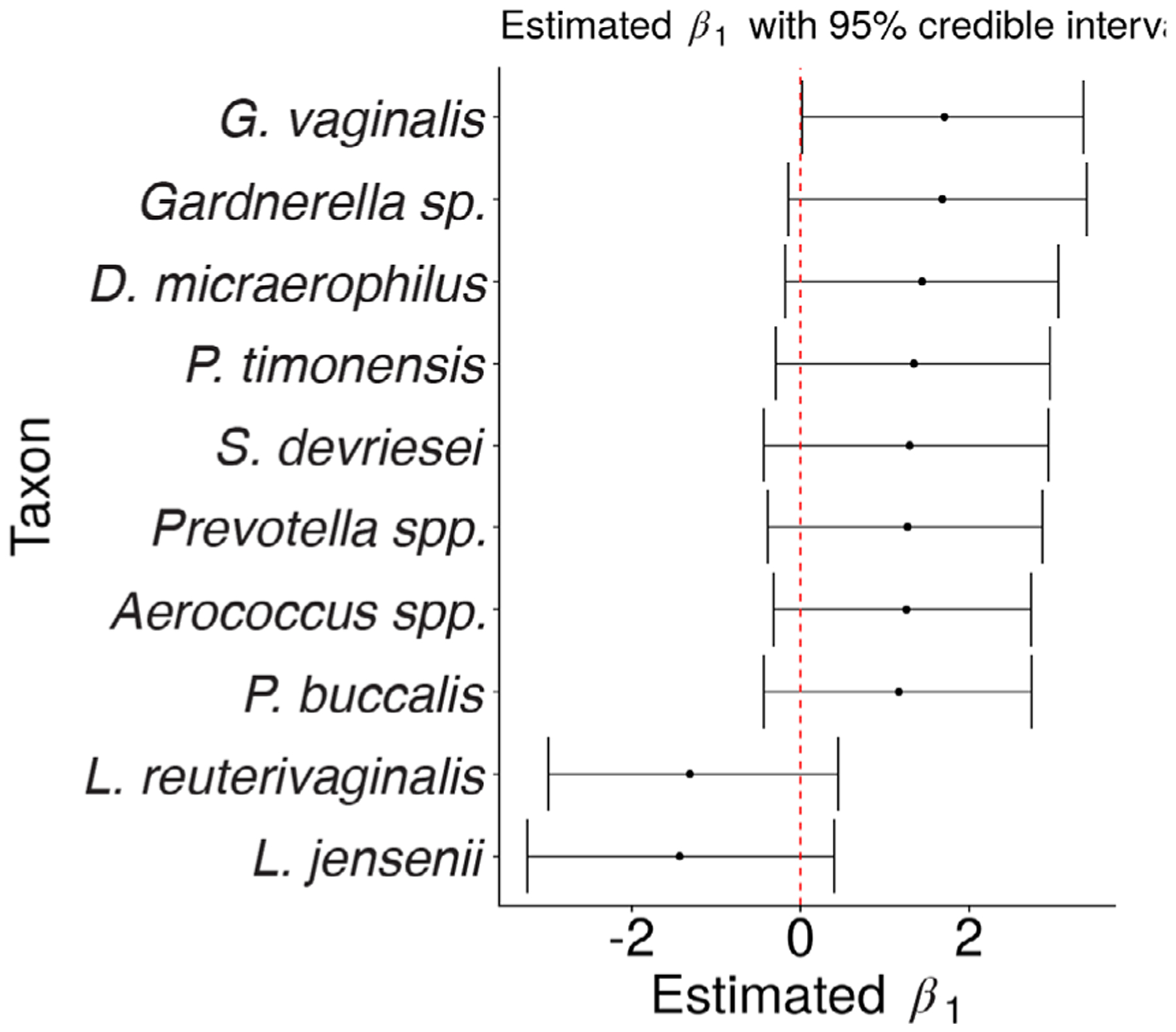
Posterior mean estimates from the proposed varying-efficiency Bayesian model of the coefficient on HIV-positive samples in the model for log concentration. The taxa with $\left|{\hat{\beta }}_{1,j}\right|$ ranked in the top 10 among all taxa are shown. A total of 95% credible intervals are displayed in the horizontal bars

Finally, Figure 5 shows point estimates and 95% credible intervals for *β*_1_ for the 10 taxa such that $\left|{\hat{\beta }}_{1,j}\right|$ is largest. For example, we find that the expected concentration of *G. vaginalis* for a HIV-positive subject from this cohort is between 1.02 and 28.6 times higher than the expected concentration of *G. vaginalis* for a HIV-negative subject from this cohort (95% credible interval). This result is consistent with the findings of Gosmann *et al*. (2017).

In the Supporting Information (Section SI 5), we also present results of a test-set analysis using the estimated parameters of both the efficiency-naïve and varying-efficiency Bayesian models based on the 55 women with-held from the primary analysis. We find that test-set prediction interval coverage varies across taxa, with mean coverage of approximately 73%.

### 5.3 | Leave-one-out analysis to predict observed qPCR

We performed a jackknife analysis to validate our proposed method on these data. In this analysis, we first restricted the data set to only those taxa with observed concentrations, leaving us with 13 taxa with both concentration and relative abundance data. Then we removed each taxon *k* ∈ {1, 2, …, 13} in turn from the observed qPCR matrix, computed the three estimators of *μ*_*ik*_ (naïve; efficiency-naïve; and varying-efficiency) and predictions for *V*_*ik*_, as well as prediction intervals for *V*_*ik*_. We then calculated mean squared prediction error and average coverage of prediction intervals (averaging over *i* = 1, …, 55), comparing the estimates of concentration to the observed qPCR concentration.

**FIGURE 6:**
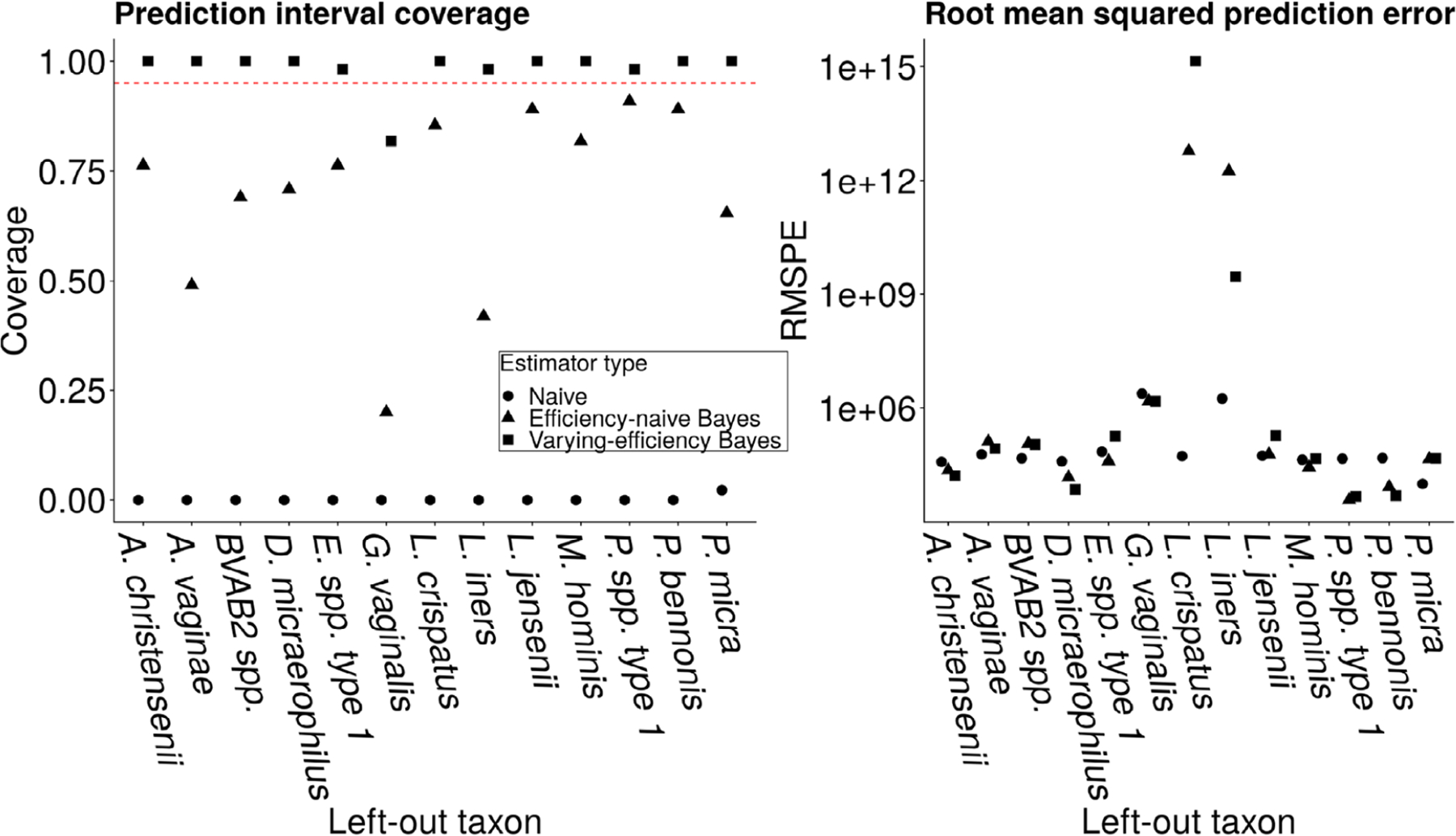
Left: Average coverage of nominal 95% prediction intervals (Wald-type intervals) for the left-out taxon averaged over study participants. Right: MSPE on the left-out taxon. Circles denote the naïve estimator, triangles denote the efficiency-naïve Bayesian estimator, and squares denote the proposed varying-efficiency Bayesian estimator

Figure 6 displays the prediction interval coverage and MSPE for the left-out taxon. Prediction interval coverage of the proposed varying-efficiency estimator is at or higher than nominal for 12 of 13 left-out taxa. Furthermore, for 11 of 13 left-out taxa, the RMSPE is comparable across the three estimators. When either *L. crispatus* or *L. iners* is left out, both hierarchical models have higher RMSPE than the naïve method, even though the coverage of the variable-efficiency method is controlled when these taxa are omitted. In contrast, neither efficiency-naïve approach controls coverage when these taxa are omitted. *L. crispatus* or *L.iners* have the highest conditional mean relative abundance in the subcomposition of taxa for which qPCR data are available (these taxa correspond to the two *j* that maximize $\frac{\sum_{i=1}^{n}W_{ij}I\left\{W_{ij}>0\right\}/M_{i}}{\sum_{i=1}^{n}I\left\{W_{ij}>0\right\}}$ among taxa *j* = 1, …, *q*^obs^), suggesting that having qPCR data for taxa that, when present, are present in high abundance may particularly improve the accuracy of *V*_*ij*_ predictions.

**FIGURE 7:**
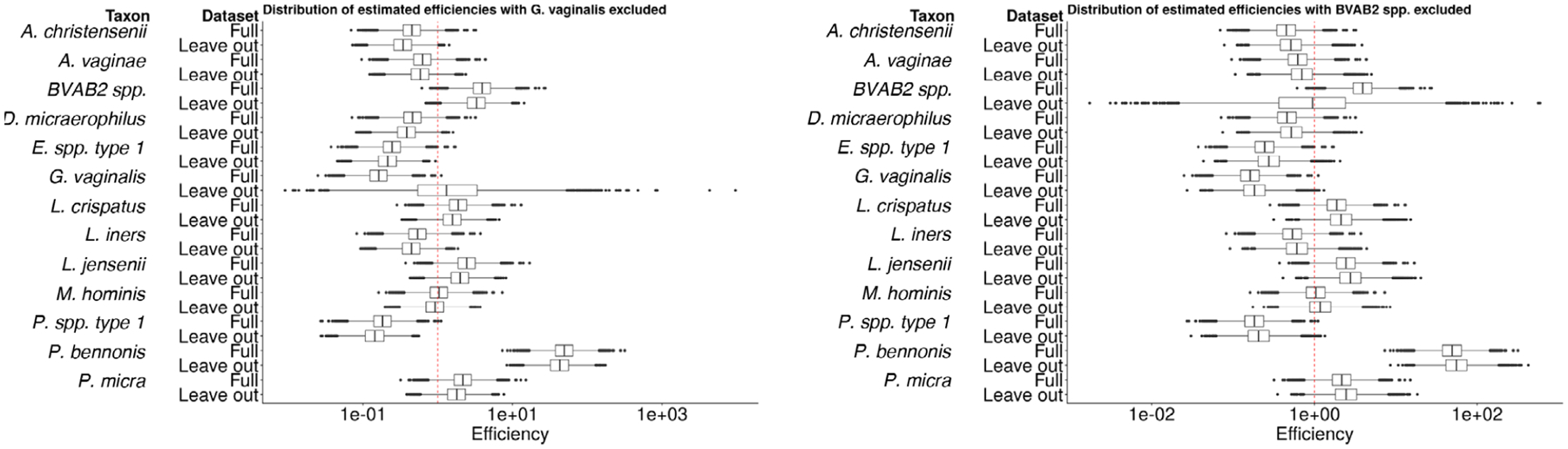
Boxplots showing the posterior distribution of estimated efficiencies. Left: estimated efficiencies from the full data analysis and from an analysis where *G. vaginalis* was left out. Right: estimated efficiencies from the full data analysis and from an analysis where *BVAB2* spp. was left out

We conclude by investigating the robustness of the estimators of efficiency to the inclusion of additional qPCR data. In Figure 7, we contrast the distribution of the estimated efficiencies in an analysis with all 13 taxa (the full-data analysis) against an analysis with a taxon left out. In the left-hand panel, we leave out *G. vaginalis*; in the right-hand panel, we leave out *BVAB2 spp*. We see in the left-hand panel that the distributions of efficiency for all taxa are nearly identical between the leave-one-out analysis and the full-data analysis, except that the distribution of *G. vaginalis* regresses to the mean and increases in variance when that taxon is left out. This indicates that *G. vaginalis* is a low-efficiency taxon. Note that the median estimated efficiency is close to the prior mean value in the leave-one-out analysis. We see the same pattern of regression to the mean and increase in uncertainty when *BVAB2* spp is left out. The inclusion of *BVAB2* spp., which is a high-efficiency taxon, alters the estimated efficiencies of the remaining taxa, resulting in increased estimated variance in many cases. These results indicate that the algorithm learns differently based on which taxa are observed: if a taxon with an extreme efficiency (e.g., in these data *BVAB2*spp. has a very high efficiency) is observed in both the absolute and relative abundance data, then the algorithm detects this larger variance in the efficiencies. This reinforces that even a model designed to account for the distribution of varying efficiencies cannot accurately predict the efficiency of an individual taxon when only relative abundance data are available. Note that these findings corroborate existing literature: Tettamanti Boshier *et al*. (2020) found that *BVAB2* spp. is a high-efficiency taxon, and McLaren *et al*. (2019) found that *G. vaginalis* is a low-efficiency taxon.

## 6 | DISCUSSION

In this paper, we developed a statistical procedure for jointly modeling absolute and relative abundance data, with a specific application to qPCR and 16S data collected on microbial communities. We proposed a hierarchical model with the following appealing characteristics: (i) point and interval estimators for the true and realized absolute abundances can be constructed for all taxa and all samples; (ii) average coverage of credible and prediction intervals is controlled at or above the nominal level; (iii) the efficiency of taxon detection of the different technologies is explicitly modeled and allowed to vary; and (iv) the method is implemented as an open-source R package. To our knowledge, our proposed hierarchical model is the first statistical model for this microbial multiview data structure.

We found strong evidence for differing efficiency of taxon detection between qPCR and 16S technologies. Given that the collection of qPCR data involves calibration (via a “standard curve”) and 16S relative abundance data does not usually involve any calibration, we modeled the efficiency of the 16S data compared to the qPCR data, rather than the opposite approach. This is consistent with recent literature (McLaren *et al*., 2019). Our method can also be used with other technologies for obtaining absolute and relative abundance data. For example, data from plate counting or flow cytometry could replace qPCR data, and a different taxonomically informative marker could replace 16S sequencing. Regardless of the technologies used, the default parameters in our software should be adjusted to reflect the units and scale of the data under study.

Empirically, we found that modeling the efficiency of the different technologies is critical for obtaining accurate estimates of microbial abundance. Tettamanti Boshier *et al*. (2020) found that a naïve approach consistently overestimates the concentration of certain taxa by an order of magnitude (e.g., *BVAB2*). In a leave-one-out approach, we observed that modeling varying efficiency achieves near-nominal coverage of prediction intervals, while failing to model varying efficiency does not control coverage (Figure 6). Variation in efficiency between taxa implies that while our method controls coverage on average across all taxa, these properties are not guaranteed for each individual taxon. Incorporating uncertainty in efficiencies results in wider intervals for the true microbial concentration, but because coverage is controlled, it accurately reflects the level of uncertainty in estimating absolute abundance. We believe that modeling efficiency is a significant advantage of our method over other proposals in the literature for combining relative and absolute abundance data.

One advantage of both the proposed method and choice of the Stan modeling software is that the hierarchical model can be easily customized to accommodate different experimental designs, prior distributions, and models for the data. For example, if the analyst prefers a Negative Binomial distribution for *V*_*ij*_ over the default choice of a Poisson distribution, this can be easily substituted; it is also easy to substitute a different model choice for *W*_*i*·_ (e.g., Dirichlet-multinomial or log-multivariate normal). Similarly, if the analyst is considering an analysis of 16S samples obtained from multiple batches, then efficiency parameters could depend on the batch and the taxon. That is, if *i* indexes the sample, *j* indexes the taxon, and *k* indexes the batch, the efficiencies could be modeled as $e_{jk}\sim \text{Lognormal}\left(\xi_{j},\sigma_{\xi }^{2}\right)$ and $\xi_{j}\sim \text{Lognormal}\left(0,\sigma_{e}^{2}\right)$ in order that each taxon’s efficiency in each batch can vary around an overall efficiency for that taxon. We have provided examples at statdivlab.github.io/paramedic illustrating how to implement these customizations.

It is possible to integrate the results of our method into a downstream analysis (e.g., an analysis incorporating *V* and/or *μ* along with additional data sources) via multiple imputation by sampling from the posterior distribution of *V*. Alternatively, an inverse-variance weighted analysis of *μ* could be performed. That is, while our illustration of the method in Section 5 reflected the data and focus of McClelland *et al*. (2018), the posterior distributions of the parameters of our model could be used in a variety of settings.

In the absence of covariate data, our method involves estimating *n* × *q* concentration parameters *μ*_*ij*_ and *q* efficiency parameters *e*_*j*_. The inclusion of additional samples therefore increases the number of parameters to estimate [a Neyman-Scott problem (Neyman and Scott, 1948)]. In addition, for small *q*^obs^ the prior distribution on the efficiencies will play a large role in determining the width of interval estimates for the concentrations *μ*_*ij*_. For these two reasons, instead of increasing *n* or *q*, *q*^obs^ should be increased where possible to reduce interval width (see Figure 2). Varying the prior parameters *α*_*σ*_ and *κ*_*σ*_ also alters the width of intervals (see SI Figure 3.3). Future modeling work could model the correlation structure between taxa (see Gibson and Gerber, 2018); remove the restriction that qPCR data must be available for all *q*^obs^ taxa for all samples; and use additional data on the total bacterial load, $\sum_{j=1}^{q}V_{ij}$, to improve estimates of *μ* and *V* using our proposed varying efficiency model.

The major limitation of our method is its computational burden. While our method is less time-intensive than developing new qPCR primers (which can take months and thousands of dollars of laboratory equipment and supplies), our method may run for a week or more, depending on *n*, *q*, and *q*^obs^. As a result, the gains in coverage of credible and prediction intervals come at the expense of computation time. We also noticed diminished interval coverage on a test data set. While we may obtain good posterior estimates of some taxon-level parameters (e.g., *β*_0_ and *β*_1_) using our procedure, the taxon-specific efficiency is difficult to transfer to new data; additionally, the true concentrations *μ*_*ij*_ are inherently difficult to predict due to the individual-level variation present in these data. For these reasons, we advocate running the analysis on all participants in a study in practice.

The proposed method provides a general approach for jointly modeling absolute and relative abundance data where each taxon’s detection efficiency differs across the two data sources. Note that our approach to modeling efficiency can model any multiplicative scaling factor between the data sources, including gene copy number. However, our motivating data sources were 16S community profiling and taxon-specific qPCR targeting the 16S gene. Because both methods targeted the same gene, our efficiency estimators are not estimating 16S copy number. In the case that different amplicons are targeted and copy numbers are known, copy number differences could be explicitly included with a minor modification to our proposed procedure.

## Supplementary Material

Supporting Information

## Data Availability

The data that support the findings of this paper are available on request from the authors. The data are not publicly available due to privacy or ethical restrictions. Any request for data must include written approval for the proposed analysis from the Kenyatta National Hospital – University of Nairobi Ethics and Research Committee. Application forms and guidelines can be accessed at https://erc.uonbi.ac.
