# Supplementary material for: A multiview model for relative and absolute microbial abundances
Source: Biometrics. 2021 Jun 8;78(3):1181–94. doi: 10.1111/biom.13503 (PMC8982138; doi:10.1111/biom.13503)
Supplement: Supplementary file 1 — Web Appendices, Tables, and Figures referenced in Sections 3, 4, and 5 are available with this paper at the Biometrics website on Wiley Online Library. The methods proposed in this manuscript are all implemented in the R package paramedic (Predicting Absolute and Relative Abundance by Modeling Efficiency to Derive Intervals and Concentrations) available at github.com/statdivlab/paramedic. All experiments and data analyses may be replicated using code available at github.com/bdwilliamson/paramedic_supplementary; the code is also available with this paper at the Biometrics website on Wiley Online Library. [file BIOM-78-1181-s002.pdf]

Supporting Information for "A multi-view model for relative and absolute microbial abundances"

Brian D. Williamson, James P. Hughes, Amy D. Willis

1 Derivation of interval estimates

1.1 Interval estimates based on the naïve estimator

Suppose that we observe data W_{ij} for $i = 1, \ldots, n$ and $j = 1, \ldots q$ and V_{ij} for $i = 1, \ldots, n$ and $j = 1, \ldots, q^{\text{obs}}$, where $q^{\text{obs}} < q$. Suppose the data is generated according to the following model:

$$\begin{aligned} V_{ij} \mid \mu_{ij} &\sim Poisson(\mu_{ij}), \\ p_{ij} &= \frac{\mu_{ij}}{\sum_{\ell=1}^{q} \mu_{i\ell}}, \\ W_{i\cdot} \mid M_i, \mu_{i\cdot} &\sim Multinomial(M_i, p_{i\cdot}). \end{aligned} \tag{S1}$$

Based on model (S1), for each sample $i = 1, \ldots, n$ we have the relationship

$$\frac{\mu_{ij}}{\sum_{j=1}^{q^{\text{obs}}} \mu_{ij}} = \frac{p_{ij}}{\sum_{j=1}^{q^{\text{obs}}} p_{ij}},$$

where $p_{ij} = \mu_{ij} / \sum_{j=1}^{q} \mu_{ij}$. This relationship suggests the naïve estimator

$$\begin{aligned} \hat{\mu}_{ij}^{\text{naïve}} &:= V_{ij} \text{ for } j = 1, \ldots, q^{\text{obs}} \\ \hat{\mu}_{ij}^{\text{naïve}} &:= \frac{\sum_{j=1}^{q^{obs}} V_{ij}}{\sum_{j=1}^{q^{obs}} W_{ij}} W_{ij} \text{ for } j = q^{\text{obs}} + 1, \ldots, q. \end{aligned} \tag{S2}$$

Simple algebraic manipulations yield that, for each $i = 1, \ldots, n$,

$$\begin{aligned} \text{Var}(\log \hat{\mu}_{ij}) = {} & \text{Var}\left(\log \sum_{j=1}^{q^{\text{obs}}} V_{ij}\right) + \text{Var}(\log W_{ij}) + \text{Var}\left(\log \sum_{j=1}^{q^{\text{obs}}} W_{ij}\right) \\ & - 2\text{Cov}\left(\log W_{ij}, \log \sum_{j=1}^{q^{\text{obs}}} W_{ij}\right). \end{aligned} \tag{S3}$$

We then use the delta method and simplify to obtain each individual term in (S3):

$$\begin{aligned}
\mathrm{Var}\left(\log \sum_{j=1}^{q^{\mathrm{obs}}} V_{ij}\right) &= \frac{1}{\sum_{j=1}^{q^{\mathrm{obs}}} \mu_{ij}}; \\
\mathrm{Var}(\log W_{ij}) &= \frac{1-p_{ij}}{M_i p_{ij}}; \\
\mathrm{Var}\left(\log \sum_{j=1}^{q^{\mathrm{obs}}} W_{ij}\right) &= \frac{1-\sum_{j=1}^{q^{\mathrm{obs}}} p_{ij}}{M_i \sum_{j=1}^{q^{\mathrm{obs}}} p_{ij}}; \text{ and} \\
\mathrm{Cov}\left(\log W_{ij}, \log \sum_{j=1}^{q^{\mathrm{obs}}} W_{ij}\right) &= -\frac{1}{M_i}.
\end{aligned}$$

Setting $\hat{p}_{ij} = W_{ij}/M_i$, we have that

$$\widehat{\mathrm{Var}}(\log \hat{\mu}_{ij}) = \frac{1}{\sum_{j=1}^{q^{\mathrm{obs}}} V_{ij}} + \frac{1-\hat{p}_{ij}}{M_i \hat{p}_{ij}} + \frac{1-\sum_{j=1}^{q^{\mathrm{obs}}} \hat{p}_{ij}}{M_i \sum_{j=1}^{q^{\mathrm{obs}}} \hat{p}_{ij}} + \frac{2}{M_i}.$$

Based on these results, model (S1), and an additional application of the delta method, the prediction variance for V_{ij} is given by

$$\widehat{\mathrm{Var}}(\log V_{ij}) = \frac{1}{\hat{\mu}_{ij}} + \widehat{\mathrm{Var}}(\log \hat{\mu}_{ij}).$$

1.2 Quantile-based prediction intervals for abundance

In Section 3.2.2 (main text) we described a procedure for constructing Wald-type prediction intervals for V_{ij}. We also investigated the performance of two approaches to constructing quantile-based prediction intervals for V_{ij}, which we now describe.

The first approach is calculated by taking $\mu_{ij}^{\alpha/2}$ and $\mu_{ij}^{1-\alpha/2}$, the $\alpha/2$ and $1-\alpha/2$ quantiles of the sampling distribution of μ_{ij}, respectively; then taking the $\alpha/2$ quantile of the $Poisson(\mu_{ij}^{\alpha/2})$ distribution as the lower prediction interval limit and the $1-\alpha/2$ quantile of the $Poisson(\mu_{ij}^{1-\alpha/2})$ distribution as the upper prediction interval limit. We call this approach *credible interval-based* because the $\alpha/2$ and $1-\alpha/2$ quantiles of the sampling distribution of μ_{ij} form a $(1-\alpha) \times 100\%$ credible interval for μ_{ij}.

The second approach, which we call *sampling distribution-based*, is calculated by generating $V_{ijb} \sim Poisson(\mu_{ijb})$, where b denotes the Markov Chain Monte Carlo (MCMC) replicates of each μ_{ij}; the prediction interval is then $[V_{ijb}^{\alpha/2}, V_{ijb}^{1-\alpha/2}]$, where V_{ijb}^{q} denotes the qth quantile of $\{V_{ijb}\}_{b \geq q}$.

These two approaches to constructing quantile-based prediction intervals are similar, and are indeed connected. Both options are of the following general form: (a) For each μ_{ijb}, select $V_{ijbk} \sim Poisson(\mu_{ijb})$, $k = 1, \ldots, N$, then (b) Take the $\alpha/2$ and $1-\alpha/2$ quantiles of the V_{ijbk}. The first option is equivalent to letting $N \to \infty$; the second option is equivalent to setting $N = 1$.

We do not show performance of these intervals in the manuscript since it was very similar to

performance of the Wald-type intervals. Both types of intervals may be used with either the efficiency-naïve or varying-efficiency Bayesian estimators proposed in the main manuscript.

2 Initializing chains and assessing algorithm convergence

In the simulated examples of Section 4 (main text), we used four chains, each with 10,000 burn-in iterations and 10,500 total iterations, to fit our proposed algorithm. We ran all analyses on a high-performance computing cluster of Linux nodes each with at least four cores and 16GB of memory; each individual simulation replicate may have been allocated less memory at run-time. Rather than initializing the chains at random values, we used simple estimates of μ, β, and Σ as initial values. The naïve estimate of μ (Eqn. (4) in main text) provides a starting point for these three model parameters: we use the column means of $\log \hat{\mu}^{\text{naïve}}$ as an initial estimator of β, where zeroes in $\hat{\mu}^{\text{naïve}}$ are set to zero in $\log \hat{\mu}^{\text{naïve}}$. We use the column variances of $\log \hat{\mu}^{\text{naïve}}$ as an initial estimator of the diagonals of the diagonal matrix Σ. For the first chain, we provide an initial value for μ and initialize the other parameters randomly; for the second chain, we provide an initial value for β and initialize the other parameters randomly; for the third chain, we provide an initial value for Σ and initialize the other parameters randomly; and in the fourth chain, we initialize all parameters randomly. In any simulation where we model efficiency, we initialize σ_e randomly.

In the data analyses of Section 5 (main text), we used six chains. In these cases, we initialized the first four chains as described above, and initialized each parameter in the final two chains using random values.

We used the Gelman-Rubin $\hat{R}$ statistic and trace plots to assess algorithm convergence. Ideally, the $\hat{R}$ statistic is close to one, and trace plots show well-mixed chains after the burn-in period. We confirmed that this was the case in all simulations and data analyses.

3 Additional numerical results

Before providing any additional empirical results, we first provide the exact specification of the performance metrics we computed in all simulations:

(i) Root mean squared error for μ_{ij}, averaged across all n samples and q taxa:

$$RMSE(\widehat{\mu})_b := \sqrt{\frac{1}{nq}\sum_{i=1}^{n}\sum_{j=1}^{q}(\widehat{\mu}_{ijb} - \mu_{ijb})^2}.$$

(ii) Root mean squared prediction error (RMSPE) for V_{ij}, $j = q^{obs}+1, \ldots, q$, averaged across all n samples and $q - q^{\text{obs}}$ unobserved taxa:

$$RMSPE(\widehat{V}_{unobserved})_b = \sqrt{\frac{1}{n(q-q^{\text{obs}})}\sum_{i=1}^{n}\sum_{j=q^{\text{obs}}+1}^{q}(\widehat{V}_{ijb} - V_{ijb})^2}.$$

(iii) Average coverage of 95% posterior credible intervals for μ_{ij}: Let $(\widehat{I}^{\ell}_{ijb}, \widehat{I}^{u}_{ijb})$ be the proposed credible interval for μ_{ij} in the bth simulation. Then

$$Coverage(\widehat{\mu})_b = \frac{1}{nq}\sum_{i=1}^{n}\sum_{j=1}^{q} I(\widehat{I}^{\ell}_{ijb} \leq \mu_{ijb} \leq \widehat{I}^{u}_{ijb}).$$

(iv) Average coverage of 95% posterior prediction intervals for V_{ij}, $j = q^{obs}+1,\ldots,q$: Let $(\widehat{PI}^{\ell}_{ijb}, \widehat{PI}^{u}_{ijb})$ be the proposed prediction interval for V_{ij} in the bth simulation. Then

$$PredCoverage(\widehat{V}_{unobserved})_b = \frac{1}{n(q-q^{\text{obs}})}\sum_{i=1}^{n}\sum_{j=q^{\text{obs}}+1}^{q} I(\widehat{PI}^{\ell}_{ijb} \leq V_{ijb} \leq \widehat{PI}^{u}_{ijb}).$$

3.1 Soft- vs hard-centering for e

We considered two possible approaches to specifying the prior distribution on e (Section 3.2.1). The soft-centering approach is given by

$$\begin{aligned} e_j &\sim Lognormal(0, \sigma_e^2) \\ \sigma_e^2 &\sim InverseGamma(\alpha_\sigma, \kappa_\sigma), \end{aligned}$$

while the hard-centering approach is provided by $\tilde{e}_j \sim Lognormal(0, \sigma_e^2)$, $\sigma_e^2 \sim InverseGamma(\alpha_\sigma, \kappa_\sigma)$, and $e_j = \tilde{e}_j \Big/ \exp\left(\frac{1}{q^{obs}}\sum_{j'=1}^{q^{obs}} \log \tilde{e}_{j'}\right)$.

We investigated the difference between these two approaches in an experiment with $n = 50$, $q = 20$, and $q^{\text{obs}} = 7$. We generated data from the data-generating mechanism described in the main manuscript, with $\sigma_e \in \{0, 0.5, 1\}$, and in all cases fit our proposed varying-efficiency Bayesian estimator. We compared the results between running two separate Stan specifications of this model, each with 4 chains (each with 10,000 burn-in iterations and 10,500 total iterations per chain): the first using the soft-centering prior on e, and the second using the hard-centering prior on e. We used `R` version 3.4.3 for all analyses in this manuscript.

We present the results of this experiment in Figure S1 (figure numbers refer to the supporting information unless stated otherwise). In the top four-panel plot, we see that interval coverage for both μ and V is at or above the nominal 95% level regardless of whether e is hard- or soft-centered, and that RMS(P)E does not differ greatly between the two hierarchical models. These patterns hold as σ_e varies. Additionally, the performance at $\sigma_e = 0$ shows the performance of the varying-efficiency estimator when there is truly no varying efficiency. In the bottom plot, we see that the posterior mean log efficiency over all 20 taxa is much closer to zero for the soft-centered procedure than the hard-centered procedure; however, looking only among the qPCR-observed taxa, the posterior mean log efficiency is much closer to zero for the hard-centered procedure than the soft-centered procedure. This coincides with what we would expect, because the hard-centering approach is defined such that the efficiencies of observed taxa are normalized, while the soft-centering approach is not.

3.2 Effect of applying filters before evaluating performance

As we referenced in Section 4 of the main text, in practice, taxa observed in low abundance across all samples are typically excluded from analysis [Callahan et al., 2016]. Any filtering must be done on the observed data, since μ is unobserved. Since W is fully observed, it is nat-

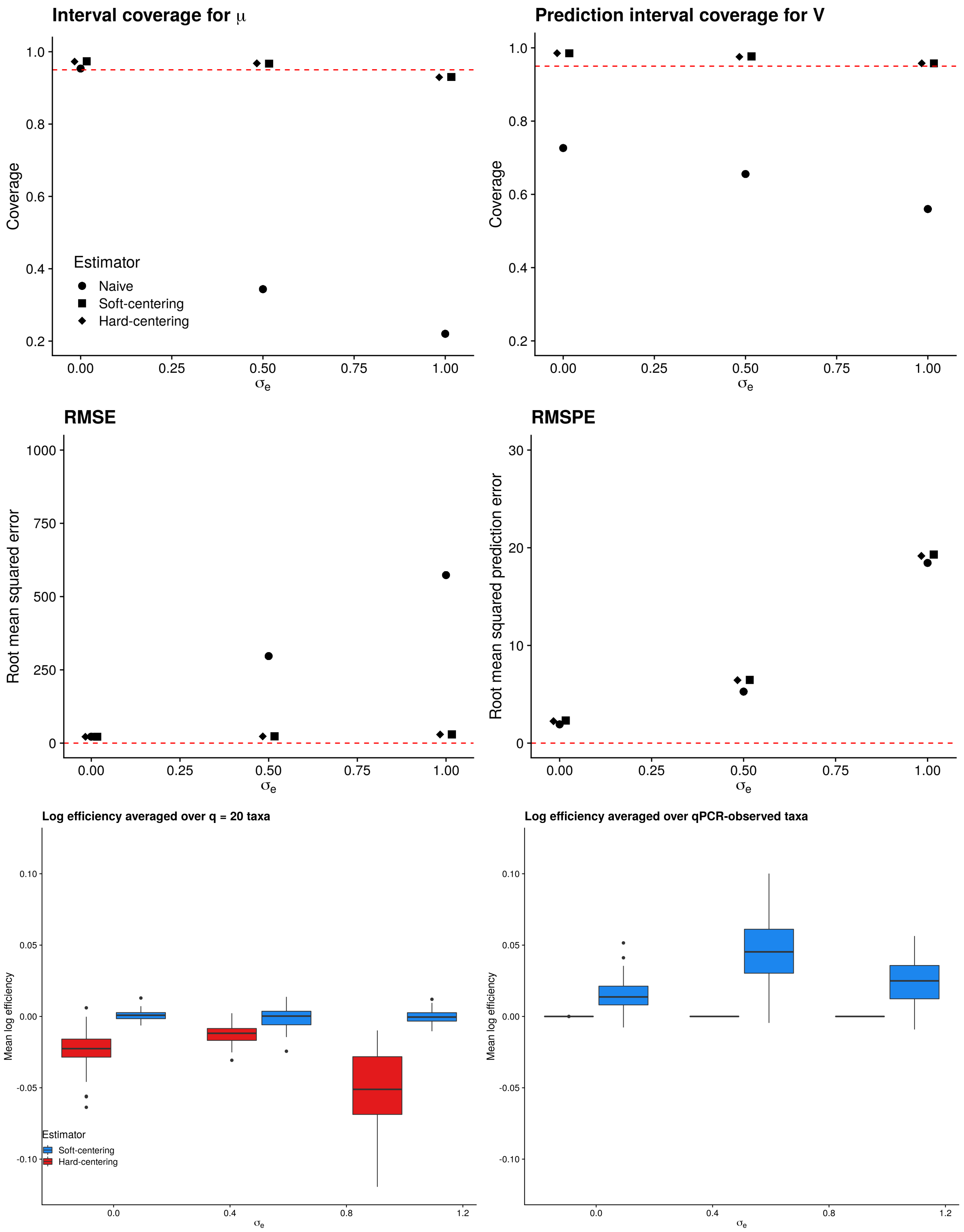

Figure S1: *Top:* interval coverage for μ and V, RMSPE, and RMSE (clockwise from top left) based on the naïve estimator (circles), varying-efficiency estimator with soft-centering (squares), and varying-efficiency estimator with hard-centering (diamonds). *Bottom:* posterior mean log efficiency averaged over all taxa (left) and only qPCR-observed taxa (right) for the soft-centered estimator (blue) vs hard-centered estimator (red).

ural that any filters used in practice would be based on W. However, in the main manuscript we chose to present results after filtering based on μ to provide a more fair comparison: using the observed data to define how the performance of a method is evaluated is somewhat nonstandard.

In practice, low abundance taxa are typically excluded because we do not expect to have much information for estimating the true concentration. Taxa for which we observe fewer counts than the number of samples appear to have particularly low information. This, in turn, will lead to poor estimates, regardless of the method used to analyze the data. The filter applied also depends greatly on the units of the observed data. These two facts suggest that a filtering rule must be developed for each dataset of interest. However, a good starting point may be that the average count for a single taxon (averaged over samples) should be greater than 1/2. This implies that there is some information, since the total count is at least half of the sample size.

To investigate whether applying different filters results in average coverage closer to the nominal level, we consider coverage of credible intervals in the simulation setting of Section 4.1 (main text). We only investigate the efficiency-naïve Bayesian estimator in this simple setting, with the expectation that the proposed varying-efficiency Bayesian estimator would perform comparably. We found that two filters performed well: only including taxon j if $n^{-1}\sum_{i=1}^{n}(W_{ij} - \frac{1}{n}\sum_{i=1}^{n} W_{ij})^2 > 1$, and only including taxon j if $n^{-1}\sum_{i=1}^{n} W_{ij} > 0.5$. Again, these filters are appropriate based on the units of data that we created in this experiment.

The results of this experiment are presented in Figure S2. In the left-hand panel, we have applied the filter on the mean of W, and see that coverage is much closer to the nominal level for $q = 40$ and $q = 60$. In the right-hand panel, we have applied a filter based on the variance of μ; in this case, coverage is not as much improved. However, filtering based on the variance of W leads to similar performance as in the left-hand panel of Figure S2.

We further investigate coverage of credible intervals in Section 3.4.

3.3 Effect of varying priors on β, Σ, and e

The prior distributions on β, Σ, and e must be specified before running the proposed algorithm. As described in Section 4 (main text), the default hyperparameters are $\sigma_\beta^2 = 50$, $\sigma_\Sigma^2 = 50$, $\alpha_\sigma = 2$ and $\kappa_\sigma = 1$. These defaults create diffuse priors on β and Σ and a less diffuse prior on e. We investigate here the effect of using both stronger and weaker priors. In one simulation, we vary the hyperparameters σ_β^2 and σ_Σ^2 in a setting without varying efficiency, and focus on the efficiency-naïve Bayesian estimator for simplicity. In a second simulation in a setting with varying efficiency, we vary the hyperparameters α_σ and κ_σ and focus on the proposed varying-efficiency Bayesian estimator. In all cases, we set $q^{\text{obs}} = 7$. In the simulation examining σ_β^2 and σ_Σ^2, we set $n = 50$. In the simulation examining α_σ and κ_σ, we set $n = 100$. In both simulations, the data was generated from the hierarchical distribution described in Section 3.2.1 (main text) with $\sigma_\beta^2 = 50$, $\sigma_\Sigma^2 = 50$, $\alpha_\sigma = 2$ and $\kappa_\sigma = 1$.

The results of the first experiment are presented in Figures S3 and S4. In Figure S3, we see that coverage is poor for small σ_β (recall that we set $\sigma_\beta = \sigma_\Sigma$); this reflects the fact that small σ_β is a strong and misspecified prior, since the true $\sigma_\beta = \sqrt{50}$. However, performance plateaus after $\sigma_\beta = \sqrt{50}$. In Figure S4, we see that the coverage results are primarily driven by the rarest taxa: for $q = 40$, we see that strong priors result in poor coverage of the rarest taxa, while diffuse priors result in the same pattern observed in the main manuscript.

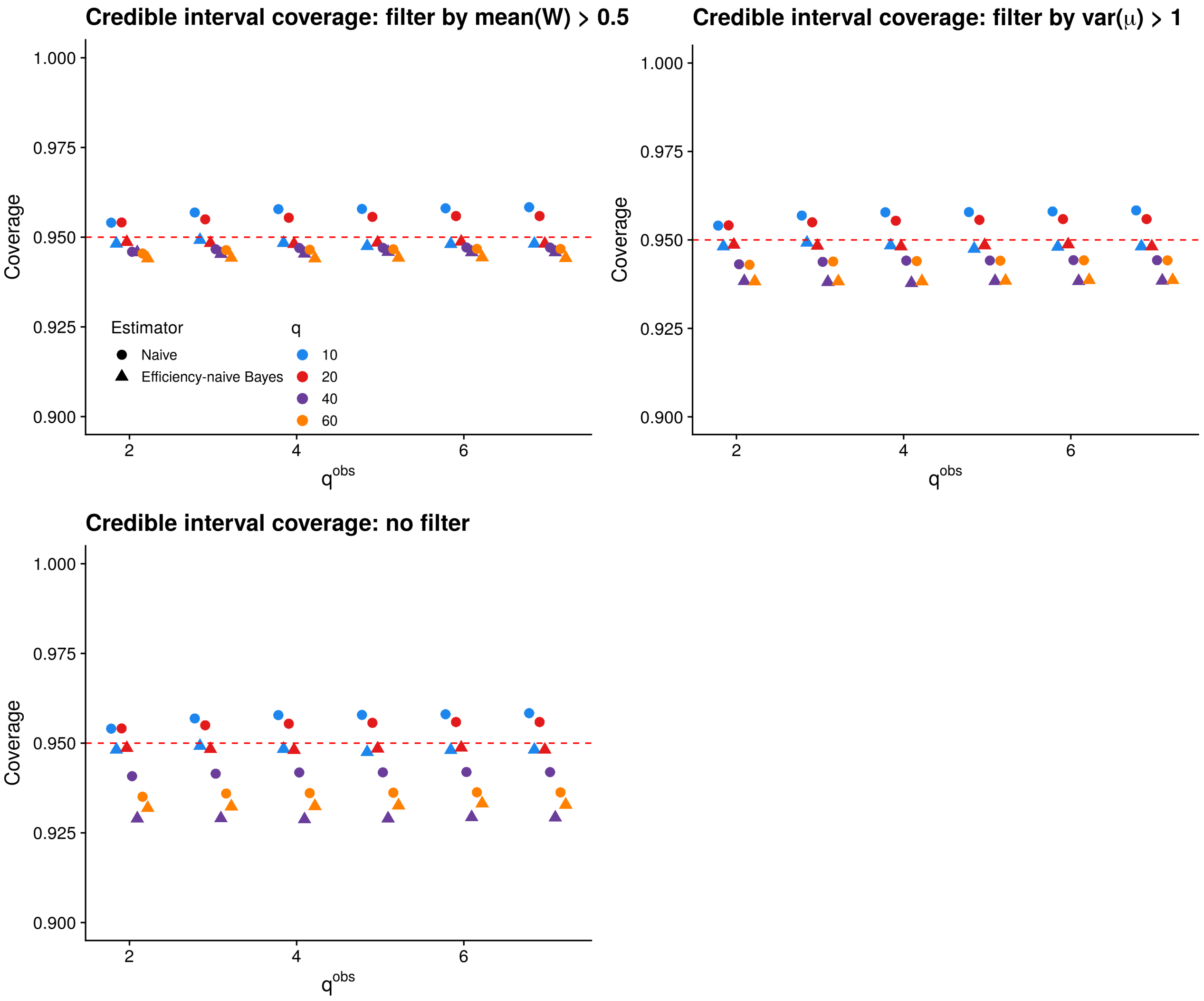

Figure S2: Coverage of nominal 95% credible intervals for μ based on the naïve estimator (circles) and efficiency-naive Bayesian estimator (triangles), in a setting with no varying efficiency (Section 4.1 of the main text), with filtering applied based on the mean of W over samples (top left) and the variance of μ over samples (top right) and no filter applied (bottom left). Recall that we cannot return an interval estimate based on the naïve estimator for (i, j) pairs where $W_{ij} = 0$. The different total numbers of taxa q are differentiated by color in the electronic version of this manuscript.

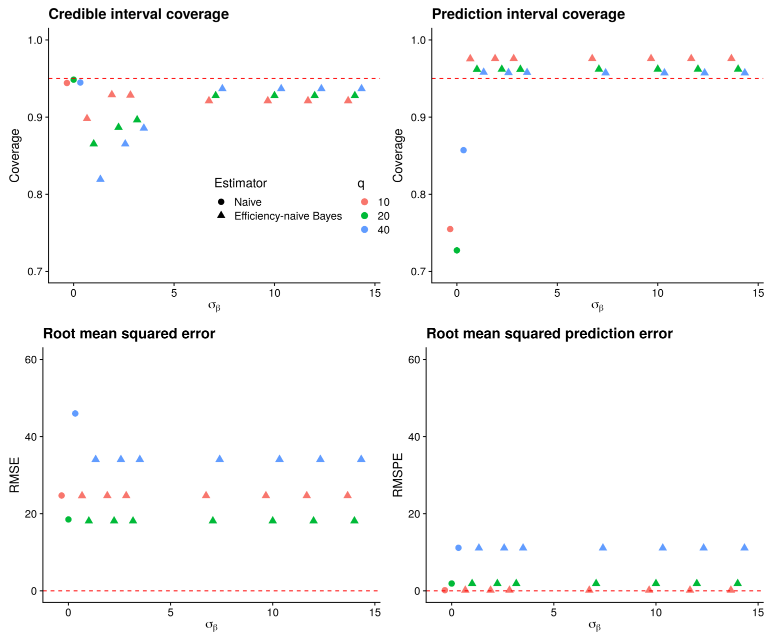

Figure S3: Performance of the naïve estimator and efficiency-naïve Bayesian estimator in a setting with no varying efficiency versus the hyperparameter σ_β for $q \in \{10, 20, 40\}$ and $q^{\text{obs}} = 7$. *Top row*: coverage of nominal 95% intervals based on the naïve (circles) and efficiency-naïve Bayesian (triangles) estimators. *Bottom row*: root mean squared prediction error and root mean squared error for both estimators. The values of q are differentiated by color in the electronic version of this manuscript.

The results of the second experiment are presented in Figure S5. In this experiment, the correct values are $(\alpha_\sigma, \kappa_\sigma) = (2, 1)$, corresponding to a mean log efficiency of 1. In the left-hand panel, we see that interval coverage for μ, V, and e is close to nominal for all choices of α_σ and κ_σ. However, as the chosen hyperparameters diverge from the data generating process, the coverage degrades. This is especially true for $(\alpha_\sigma, \kappa_\sigma) = (3, 0.5)$, which is a strongly informative prior that is centered much closer to zero than the other combinations (mean/variance is 0.25/0.125 while true mean/variance is $1/\infty$). In the middle panel, we see that the RMSE for estimating e is small for all choices of α_σ and κ_σ. In contrast, RMSE for estimating μ and V is minimized at $(\alpha_\sigma, \kappa_\sigma) = (2, 0.5)$. Finally, credible interval widths are shown in the right-hand plot. We see that a larger credible interval width (e.g., $(\alpha_\sigma, \kappa_\sigma) = (1, 0.5)$) tends to translate to a higher coverage (left-hand plot), while a smaller credible interval width (e.g., for $(\alpha_\sigma, \kappa_\sigma) = (3, 0.5)$) results in lower coverage. Taken together, these results suggest that concentrated priors reduce interval width at the possible expense of coverage. We recommend that users carefully investigate the sensitivity of their results to the chosen priors, making selections that reflect the relative importance they place on narrow intervals versus coverage.

3.4 Coverage and rank order of abundance

In Section 4.1 (main text), we investigated the effect of varying q and q^{obs} for a fixed sample size, and where there was truly no varying efficiency. We noticed that for $q = 40$ and $q = 60$, coverage of credible intervals was slightly below the nominal 95% level. To further investigate this phenomenon in the simple setting with no varying efficiency, we computed coverage across

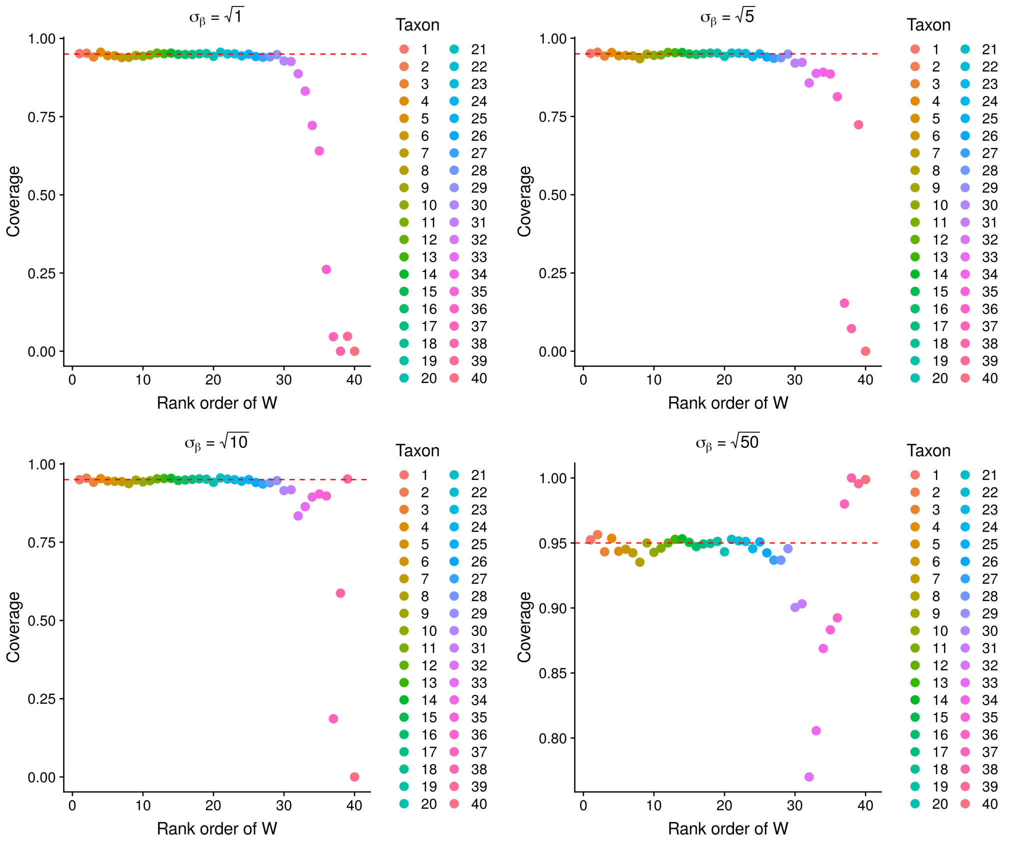

Figure S4: Credible interval coverage based on the efficiency-naïve Bayesian estimator for each taxon versus rank order in W for four different values of σ_β, in a case with no varying efficiency, $q = 40$, and $q^{\text{obs}} = 7$. Taxa are differentiated by color in the electronic version of this manuscript.

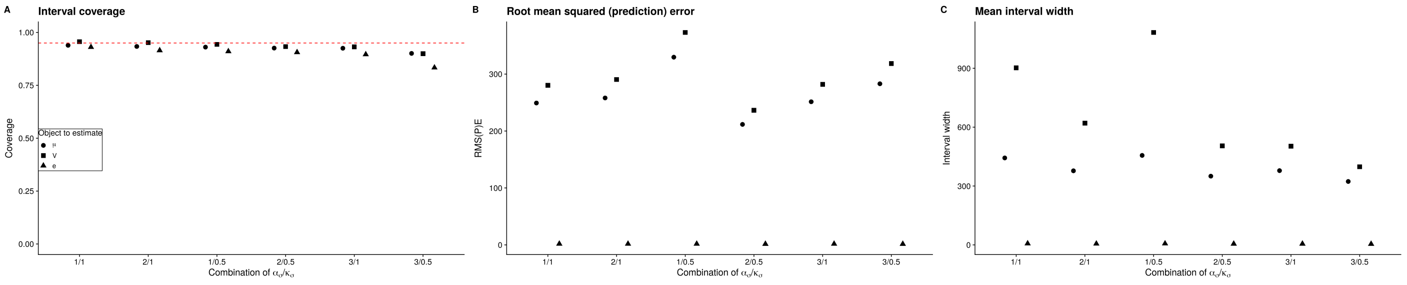

Figure S5: Credible interval coverage, root mean squared error, and interval width when estimating μ (circles), V (squares), and e (triangles), for the proposed varying-efficiency Bayesian estimator, across different values of the hyperparameters for the efficiency prior (α_σ and κ_σ). In this case, there truly is varying efficiency, $q = 40$, and $q^{\text{obs}} = 7$.

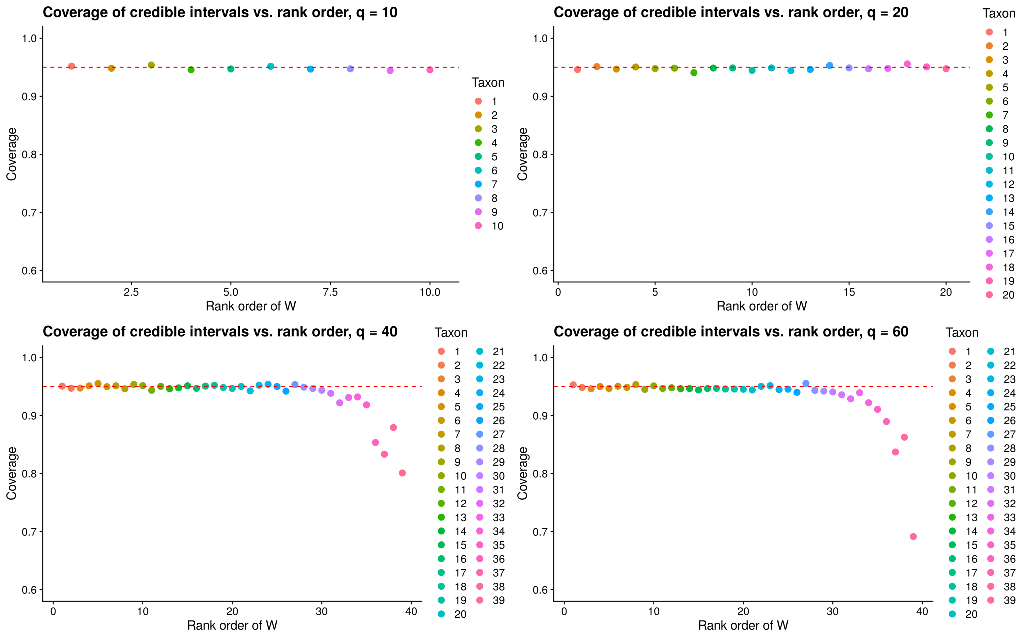

Figure S6: Coverage of nominal 95% credible intervals for μ based on the efficiency-naïve Bayesian estimator versus rank order in W, for $q \in \{10, 20, 40, 60\}$ (clockwise from top left, respectively) and $q^{\text{obs}} = 7$, in a setting with no varying efficiency (Section 4.1 of the main text). Taxon j is only included in coverage evaluation if $n^{-1} \sum_{i=1}^{n} \mu_{ij} > 1$, and the taxa are differentiated by color in the electronic version of this manuscript.

samples for each taxon and each Monte Carlo iteration. We then plotted this coverage versus the taxon's rank order in W.

The results of this investigation are presented in Figures S6 and S7, where Figure S6 is filtered to include taxon j only if $n^{-1} \sum_{i=1}^{n} \mu_{ij} > 1$ and Figure S7 is not filtered. Here, we see that for each q, there is some variability about the nominal 95% line. We also see that coverage is near the nominal level for many taxa, regardless of q. However, for $q = 40$ and $q = 60$, we see that coverage dips below the nominal level and that there is a large amount of variation for taxa with rank order greater than 34. Additionally, for $q = 60$, we see that coverage increases again for taxa with rank order greater than 40; for the lowest abundance taxa, coverage is near one.

This investigation led to a second question: do the taxa for which we have poor coverage depend on M, the read depth? We studied this by running 50 replicates of this simulation for $q = 60$ and $q^{\text{obs}} = 7$, but with $M \sim DiscreteUniform(10^3, 10^4)$ to yield smaller read depths. In Figure S8, we see that the taxa with poor coverage have shifted to a higher rank order, indicating that read depth does influence the coverage of our proposed method. This matches with our expectations: shorter read depth leads to more taxa that are observed in low abundance, which leads to decreased coverage.

Taken together, these results suggest that our method provides coverage control for high- and moderate-abundance taxa and for very rare taxa. However, our method does not provide coverage control for rare (but not very rare) taxa. One explanation for this phenomenon is our Bayesian approach: we control average coverage, but we have no guarantee to control coverage for each individual taxon. Additionally, we are using here a frequentist evaluation metric. Low coverage for somewhat rare taxa may also be explained as a logical result of the input data: for somewhat rare taxa, W is near zero for all samples (in fact, the variance of W across

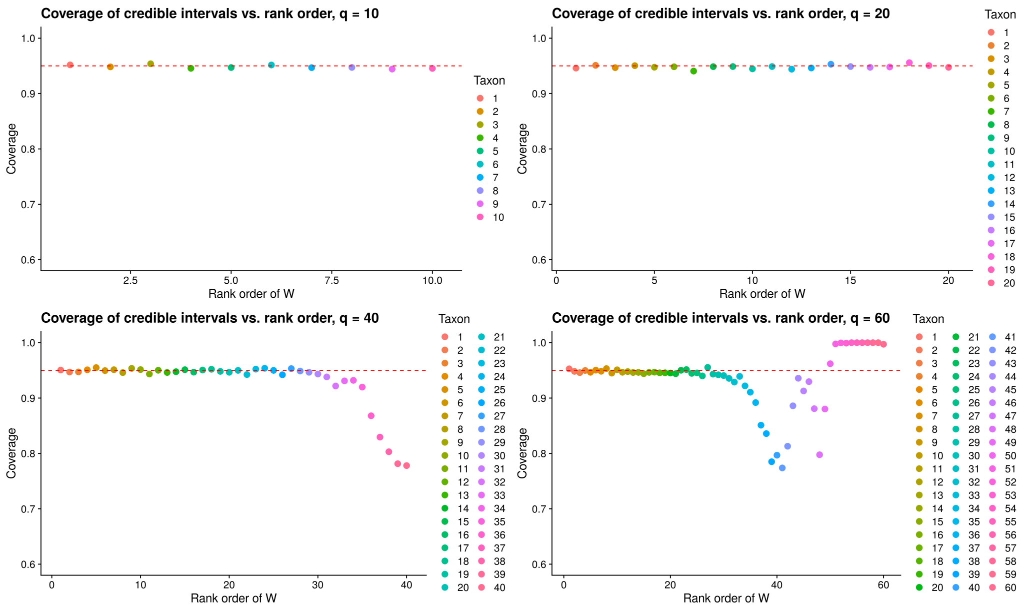

Figure S7: Coverage of nominal 95% credible intervals for μ based on the efficiency-naïve Bayesian estimator versus rank order in W, for $q \in \{10, 20, 40, 60\}$ (clockwise from top left, respectively) and $q^{\text{obs}} = 7$, in a setting with no varying efficiency (Section 4.1 of the main text). No filtering is performed prior to evaluation. The taxa are differentiated by color in the electronic version of this manuscript.

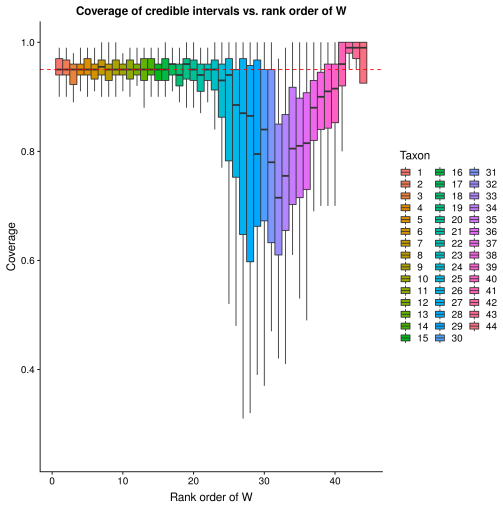

Figure S8: Coverage of nominal 95% credible intervals for μ based on the efficiency-naïve Bayesian estimator versus rank order in W, for $q = 60$ and $q^{\text{obs}} = 7$ and M ranging from 10^3 to 10^4, in a setting with no varying efficiency (Section 4.1 of the main text). The boxplots show Monte Carlo error over the 50 replications, and the taxa are differentiated by color in the electronic version of this manuscript.

samples tends to be lower than one). Since our proposed method does not borrow information across taxa, we do not expect to perform well for taxa that are somewhat rare. For extremely rare taxa, however, we have high coverage because our method essentially over-estimates the variance.

3.5 Mean squared error decoupled into bias and variance

In Section 4 (main text) we presented results in terms of mean squared error. However, it is also of interest to investigate whether or not the proposed estimator is biased.

We first investigate the average estimation bias of the efficiency-naïve Bayesian estimator in the case where there truly is no varying efficiency (Section 4.1 of the main text); this simple case provides us with a baseline to compare against our proposed varying-efficiency Bayesian estimator. In Figure S9, we show this bias only for $q^{\text{obs}} = 7$; we see similar patterns for all other values of q^{obs}. For each $q \in \{10, 20, 40, 60\}$, we show bias versus rank order in W. This allows us to see patterns in over- or under-estimation. Here, we see that the efficiency-naïve Bayesian estimator has small bias that decreases with rank order. However, as rank order increases, the true concentration decreases towards zero. Therefore, it is somewhat unsurprising that bias decreases with rank order. Of more interest is the observation that for high- and moderate-abundance taxa, the efficiency-naïve Bayesian estimator does not uniformly over- or under-estimate the true abundance. The small bias observed here suggests that the variance of the efficiency-naïve Bayesian estimator is driving mean squared error.

In Figure S10, we show the average squared estimation bias of the naïve estimator, the efficiency-naïve Bayesian estimator, and the proposed varying-efficiency Bayesian estimator in the case where there truly is varying efficiency (Section 4.2 of the main text). Here, we see that for $\sigma_e = 0$, the naïve estimator and efficiency-naïve Bayesian estimator have comparable bias for many taxa, as we saw in Figure S9; for the highest-abundance taxa in this case with no varying efficiency, the efficiency-naive and varying-efficiency Bayesian estimators are nearly identical. The proposed varying-efficiency Bayesian estimator tends to under-estimate other high abundance taxa, especially when the true variance of the efficiencies is small. As σ_e increases, we see that the naïve estimator and efficiency-naïve Bayesian estimator both have substantially more bias than the proposed varying-efficiency Bayesian estimator for high-abundance taxa. For low abundance taxa, all three estimators have similar bias. While the magnitude of bias observed in this example is larger than that in Figure S9, it is small compared to the mean squared error seen in the main manuscript, suggesting that variance again plays a large role in driving mean squared error.

3.6 Effect of model misspecification

The simulation results presented in Section 4 of the main manuscript are all in cases where the distributions for μ, e, V, and W are correctly specified. In this section we investigate the performance of our estimator under various misspecified data generating processes. Throughout this section, we simulate data from varying efficiency models (i.e., such that $Var(e_j) > 0$).

We first investigated simulating data where the distributions for μ and/or e are misspecified but the distributions of V and W are correctly specified. We consider the following possible distributions for μ:

1. A gamma distribution: $\mu_{ij} \overset{iid}{\sim} Gamma(\alpha_{\mu,j}, \beta_{\mu,j})$, where $\alpha_{\mu,j} \overset{iid}{\sim} Gamma(0.1, 10^{-4})$ for all

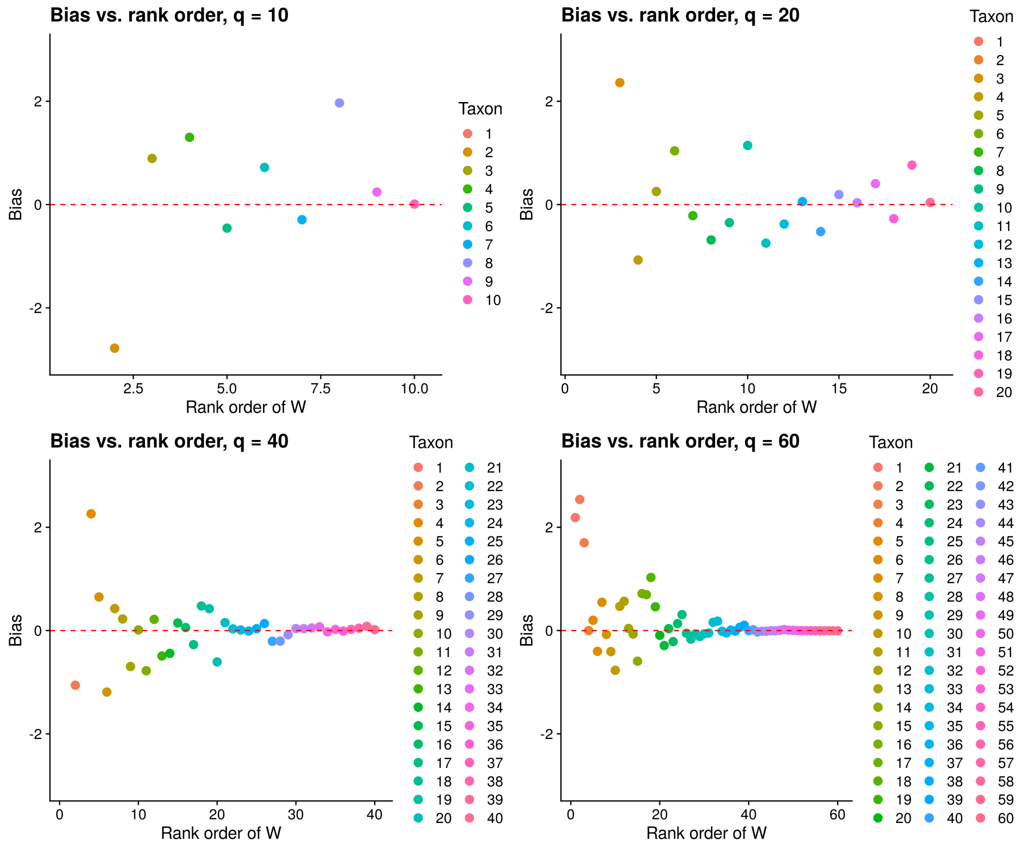

Figure S9: Estimated bias versus rank order in W for the efficiency-naïve Bayesian estimator, where $q^{\text{obs}} = 7$, in a setting with no varying efficiency (Section 4.1 of the main text). The taxa are differentiated by color in the electronic version of this manuscript.

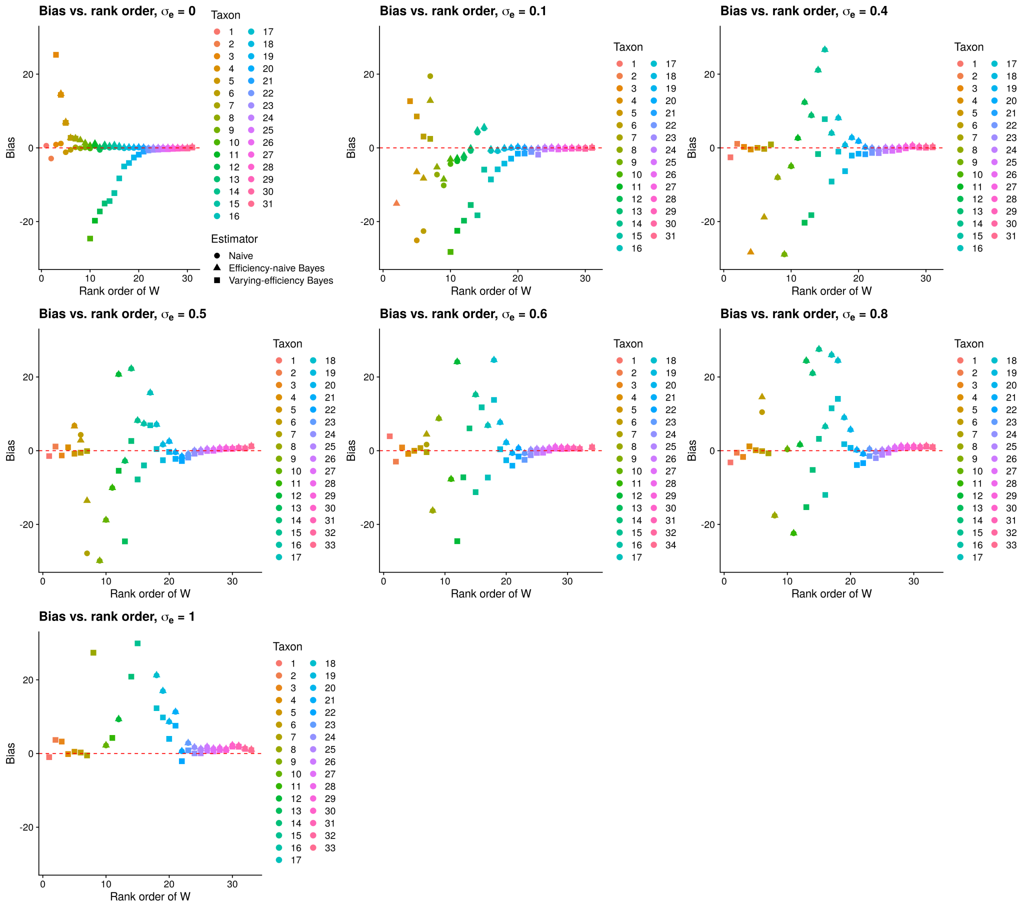

Figure S10: Estimated bias versus rank order in W for the naïve estimator (circles), efficiency-naïve Bayesian estimator (triangles), and the proposed varying-efficiency Bayesian estimator (squares), in a setting with differing levels of truly varying efficiency (Section 4.2 of the main text). The taxa are differentiated by color in the electronic version of this manuscript.

j and $\beta_{\mu,j} = 0.5$ for all j;

2. A non-central half-t distribution: $\mu_{ij} \overset{iid}{\sim} |t_2(a_{\mu,j})|$, where the non-centrality parameter is distributed as $a_{\mu,j} \overset{iid}{\sim} Uniform(0, 100)$ for all j

3. The correctly specified distribution: $\mu_{ij} \overset{iid}{\sim} N(\beta_j, \Sigma_{jj})$ where $\beta_j \overset{iid}{\sim} N(0, 50)$ and $\Sigma_{jj} \overset{iid}{\sim} Lognormal(0, 50)$ for all j

We consider the following distributions on e

1. A gamma distribution $e_j \overset{iid}{\sim} Gamma(0.5, 0.5)$ for all j

2. A half-t distribution with four degrees of freedom $e_j \overset{iid}{\sim} |t_4(0)|$

3. The correctly specified distribution: $e_j \overset{iid}{\sim} Lognormal(0, \sigma_e^2)$

We investigated the performance of our proposed approach under each combination of these true distributions on μ and e.

We also considered settings in which the distributions for μ and e are correctly specified but the distributions for V and/or W are misspecified. The distributions we considered for V are the correctly specified Poisson distribution and a negative binomial distribution:

$$V_{ij} \mid \mu_{ij}, \sigma_{V,i} \sim NegativeBinomial(\mu_{ij}, \sigma_{V,i}),$$

where $\sigma_{V,i} = 1$ for all i. The distributions we considered for W are the correctly specified multinomial distribution and a compound Dirichlet-Multinomial distribution:

$$\alpha_{i\cdot} \sim Dirichlet\left(\frac{e\mu_{i\cdot}}{\sum_{j=1}^{q} e_j \mu_{ij}}\right)$$
$$W_{i\cdot} \mid M_i, \mu_{i\cdot} \sim Multinomial(M_i, \alpha_{i\cdot}).$$

For each combination of data-generating mechanisms that we studied, we used Stan to fit our proposed varying-efficiency model using 4 chains per simulated dataset, each with 20,000 burn-in iterations and 18,000 total iterations (8,000 total iterations for each of $B = 50$ simulations for each data-generating model combination to investigate). We only display the results of this estimator and the naïve estimator; we expect that results based on the efficiency-naïve Bayesian estimator would follow similar patterns to the varying-efficiency Bayesian estimator, but would suffer from the fact that there truly was varying efficiency (similar to the main mansucript, Section 4.2). In all cases, we fix $q = 40$, $q^{\text{obs}} = 7$, and $n = 100$, and simulate M_i according to $M_i \sim DiscreteUniform(10^4, 10^5)$.

Figure S11 displays the results of this experiment (the top row shows the varying-efficiency Bayesian estimator, while the bottom row shows the naïve estimator). We denote a gamma distribution by “G”, a Poisson distribution by “P”, a multinomial distribution by “M”, a half-t distribution by “H-t”, a negative binomial distribution by “NB”, and a compound Dirichlet multinomial distribution by “CDM”. We compare all results to the correctly specified data-generating mechanism N/N/P/M. Results for N/N/P/M are shown as the left-most results in each plot.

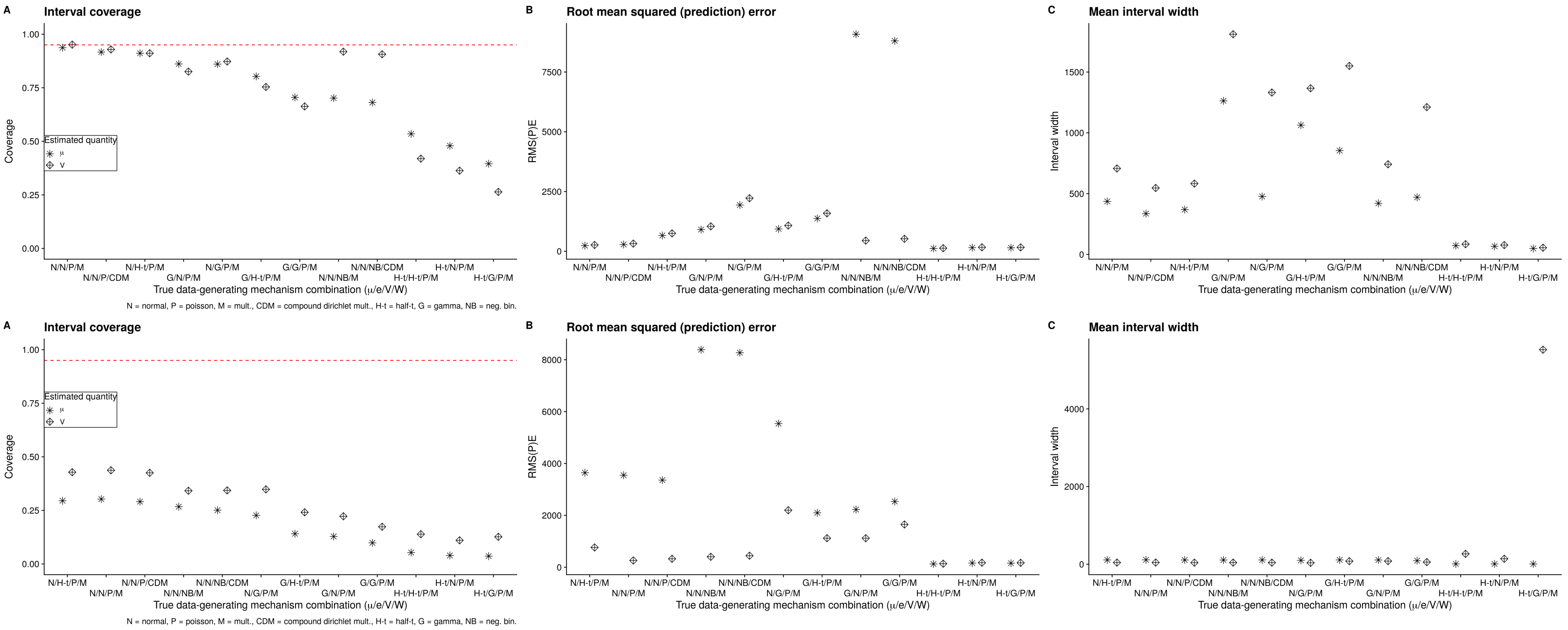

Figure S11: Performance of the proposed varying-efficiency Bayesian (top) and naïve (bottom) estimators in estimating μ (stars) and V (crossed diamonds) versus data-generating model combination for $q = 40$ and $q^{\text{obs}} = 7$ in a setting with truly varying efficiencies. (A) Interval coverage, (B) root mean squared error for μ and root mean squared prediction error for V, (C) mean interval width.

We first discuss the varying-efficiency Bayesian estimator. In the correctly specified case, coverage for μ and prediction interval coverage for V are both close to the nominal 95%. Moving from left to right, we see that coverage for both μ and V decreases slightly if the distribution for W or e alone is misspecified. If both μ and e are misspecified and μ is gamma-distributed, then coverage decreases further. If the distribution for V is misspecified, then coverage for μ suffers but coverage for V is maintained at near the nominal level. The lowest coverage for both μ and V occurs when μ follows a half-t distribution. This phenomenon appears to be driven by the adversarial nature of the half-t distribution: this is a very heavy-tailed distribution for μ. To illustrate this, we display boxplots showing the distribution of the generated objects over Monte Carlo replicates in Figure S12. The two boxplots for each data-generating mechanism combination denote whether or not V was observed for the particular taxon. We see that in the case of a noncentralized half-t distribution on μ, the observed and unobserved μ and V values are most similar across all scenarios studied here – this appears to be the main driver of the poor coverage observed in this case. In this setting there is little sparsity in V, and the highest-abundance taxa are equally likely to be in q^{obs} as the lowest abundance taxa. However, in practice, typically high abundance taxa are those for which qPCR data is available. Thus, our procedure is relatively robust to misspecifying the distribution on e, somewhat robust to mild misspecification of the model on μ, but not robust to large departures from the true data-generating distribution on μ.

The naïve estimator has consistently poor coverage, and in many cases has larger mean squared error than the propose varying-efficiency Bayesian estimator. This was expected, because there truly is varying efficiency in these experiments which the naïve estimator does not take into account.

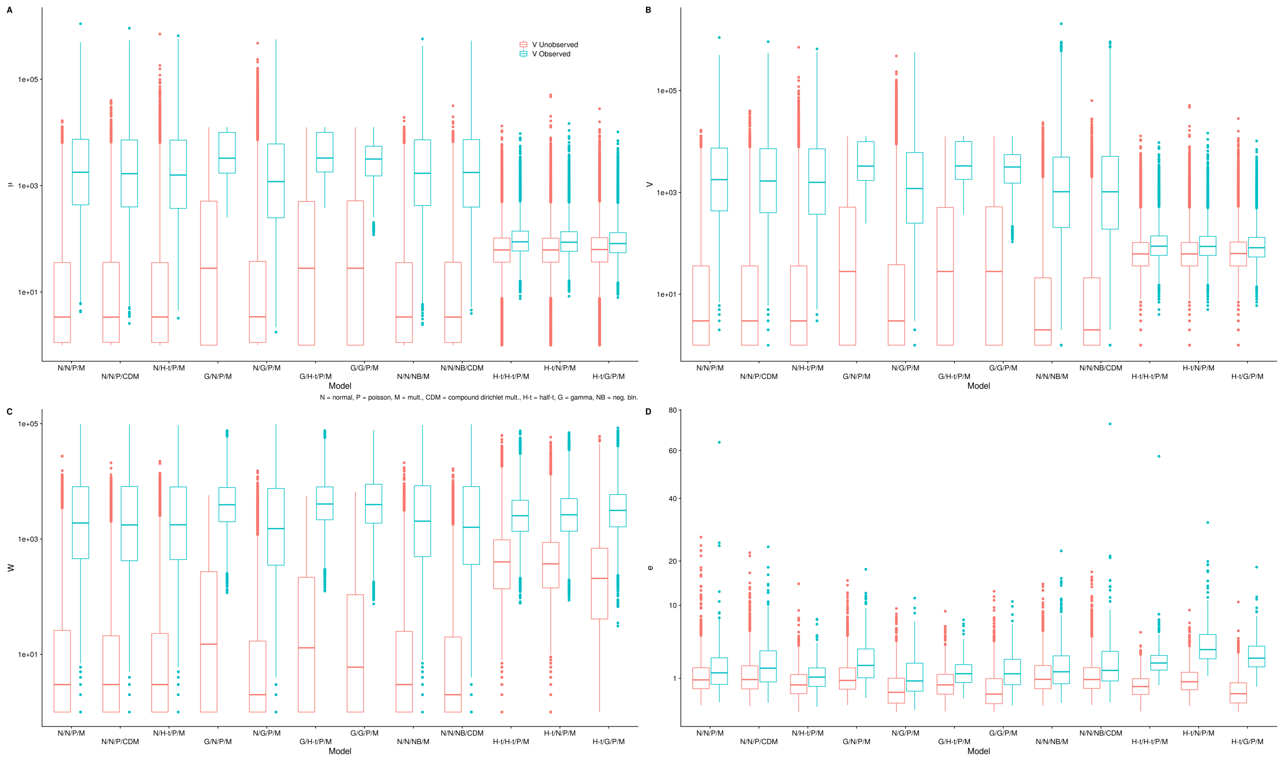

Figure S12: Boxplots showing the distribution of the true values of μ, V, e, and W (clockwise from top left) over Monte Carlo replications for each true data-generating combination in a setting with truly varying efficiencies, $q = 40$, and $q^{\text{obs}} = 7$. The distributions of each are broken up by the indicator of whether or not V_j was observed; the left-hand boxplot for each combination is the distribution for taxa 1–7 and the right-hand boxplot is the distribution for taxa 8–40.

Our procedure appears to be more robust to misspecification of W than misspecification of V (Figure S11). Coverage is near the nominal level if only W is misspecified, but if only V is misspecified coverage decreases to below 75%. This appears to be driven by large mean squared error for estimating μ for the high-abundance taxa; in contrast, prediction interval coverage is maintained at nearly the nominal level. The distribution on V here is highly misspecified: the Poisson distribution poorly approximates the negative binomial distribution with the given parameters.

4 Computational details

4.1 Stan hierarchical model specifications

As mentioned in Section 3.2 (main text), we use Stan to fit all hierarchical models. This allows substantial flexibility in how the models are specified—including specifying alternative priors to reflect knowledge about the true data-generating process and utilizing alternative parameterizations to improve model convergence. In particular, there are several methods for so-called "efficiency tuning" (Chapter 22 of the Stan User's Guide) that we have explored in an effort to achieve a reasonable limit on computation time.

The first method is by changing control parameters passed to Stan. We use the No-U-Turn Sampler (NUTS) algorithm by default; the primary parameters controlling the behavior of this algorithm (specified through the `control` argument) are `adapt_delta` (the target average proposal acceptance probability) and `max_treedepth` (controls the maximum depth of a NUTS tree). Changing these parameters may affect convergence speed, but also may affect convergence itself.

The second method is by increasing the *statistical efficiency*. For example, a well-known issue in Stan is that *centered* parameterizations can be slow to converge in some cases (see Section 22.7 of the Stan User's Guide). An example of a centered parameterization involves parameter μ and hyperparameters β and Σ:

$$\begin{aligned}\mu &\sim N(\beta, \Sigma)\\ \beta &\sim N(0,1)\\ \Sigma &\sim N(0,1).\end{aligned}$$

Indeed, hierarchical model (5) (main text) is specified in a centered form. In these settings, a *noncentered* parameterization may help to speed up convergence; for the example given above, the noncentered parameterization would place a simpler prior on a transformed parameter of μ and then back-transform:

$$\begin{aligned}\tilde{\mu} &\sim N(0,1)\\ \mu &= \sqrt{\Sigma}\tilde{\mu} + \beta\\ \beta &\sim N(0,1)\\ \Sigma &\sim N(0,1).\end{aligned}$$

We have implemented both a centered and a noncentered version of our proposed algorithm, available in `paramedic` by specifying the `centered` argument. In all cases in the manuscript, we have fit the noncentered model, and have found that it generally speeds up computation

Table 1: Time comparison (in minutes) for analyzing a dataset with n samples and q total taxa using hierarchical model (6) or (5). In all cases, $q^{\text{obs}} = 7$.

n	q	Hierarchical model	
		Efficiency-naïve (6)	Varying-efficiency (5)
100	27	86.22	200.85
100	37	111.10	324.40
200	27	359.19	550.06
200	37	301.50	1008.39

time compared to the centered model. We have also used wherever possible the log-transformed version of a distribution, which can help in computing gradients of probability distributions (especially for constrained objects, like count data).

A third method of speeding up computations is through *vectorization*. Here, the gradients of log probability functions used in the hierarchical model are made simpler by working with vectors (and fast linear algebra operations) rather than single variables or loops. Unfortunately, while some parts of our proposed hierarchical model can be vectorized (e.g., the priors on e, β_0, and Σ), others cannot—the most notable example is the counts V, which are specified as a matrix. The Poisson and Negative Binomial distributions (both of which can be specified in `paramedic`) in Stan can be used on vectors but not arrays or matrices—Stan uses strong typing, which is useful in many respects but can be limiting in others.

Finally, one could consider use a different programming method entirely. While Stan is highly flexible (e.g., allowing a swap between a Poisson and Negative Binomial distribution for V requires only a few lines of code in Stan), this flexibility can be constraining, as mentioned above. It is possible that a bespoke algorithm (e.g., a Metropolis-Hastings within Gibbs sampler) could be coded for our proposed hierarchical model (e.g., in `C++`) and could yield faster computation time. However, we believe that the benefits offered by Stan outweigh the cost of increased time, which remains less than optimizing new primers for measuring absolute abundance.

4.2 Computation time for the proposed estimators

Table 1 displays the approximate time (in minutes) to run the proposed efficiency-naïve and varying-efficiency Bayesian algorithms for 10,500 iterations (with 10,000 burn-in iterations) with six chains for $q^{obs} = 7$ and varying q and n. We ran these data analyses on a cluster computer with six cores and 256 GB of memory, using one core per chain. The chains were all initialized using random values for the model parameters. We generally observed that increasing the sample size has a larger effect on computation time than increasing the number of taxa. Additionally, the proposed varying-efficiency algorithm tends to have a longer computation time than the efficiency-naïve algorithm. One cell in the table does not fit the general trends we observed: at $n = 200$, we observed a shorter computation time for $q = 37$ than $q = 27$ for hierarchical model (6). This may be due to an anomalous load on the cluster at time of running.

5 Additional results from a study of the vaginal microbiome

In Section 5 of the main manuscript, we presented an analysis of the data from McClelland et al. [2018] where we fit the model

$$\log \mu_{i\cdot} \sim N_q(\beta_0 + \beta_1 X_i, \Sigma), \tag{S4}$$

$i = 1, \ldots, n$, where $X_i = 1$ if subject i is HIV-positive and $X_i = 0$ otherwise. In this section we present two sensitivity analyses.

In the first sensitivity analysis, we do not adjust for HIV status. That is, we fit the model

$$\log \mu_{i\cdot} \sim N_q(\beta_0, \Sigma), \tag{S5}$$

$i = 1, \ldots, n$, where $\beta_0 \in \mathbb{R}^q$. The same hyperparameters were chosen as in the original analysis (see Section 5 of the main text). Our results are shown in Figure S13. By comparing this figure to Figure 4 of the main text, we see that the estimated concentrations are extremely similar across the covariate-adjusted and unadjusted models. Interval and point estimates are available as Supplementary Data for the covariate-adjusted and unadjusted models.

We also performed a test-set analysis. After estimating the model parameters of both the efficiency-naïve and varying-efficiency Bayesian estimators on the sampled 55 women, we predicted the observed qPCR values for the held-out 55 women. It is not straightforward to obtain test-set predictions based on the naïve estimator, so we did not include this estimator in our analysis. In Figure S14, we see that while test-set prediction interval coverage varies across taxa, for many taxa the prediction interval coverage is at or above 75% (the average coverage for both estimators across taxa is approximately 73%). The mean squared error is approximately on the same order as the jackknifed mean squared error observed in the leave-one-out analysis.

We also compare our leave-one-out analysis from the covariate-adjusted analysis to our unadjusted analysis and again draw similar conclusions. In Figure S15, we see that the conclusions of the leave-one-out analysis are similar to those presented in the main manuscript (Section 5.3). In fact, prediction interval coverage is controlled for 13 out of 13 taxa (coverage was only controlled for 12 out of 13 taxa in the covariate-adjusted model). Furthermore, we tend to observe both a smaller RMSPE in the unadjusted model, along with a smaller variance in the left-out efficiencies (Figure S16).

In the second sensitivity analysis, we vary the priors on the efficiency model and fit the covariate-unadjusted model described above. In our original model, we chose $\alpha_\sigma = 4$ and $\kappa_\sigma = 3$. Here, we present results obtained from setting $\alpha_\sigma = 2$ and $\kappa_\sigma = 3$ (a more diffuse prior on the efficiency parameters). The results are shown in Figure S17. We see that varying the priors on the efficiency model results in nearly identical point estimates of concentration to both the adjusted analysis (Figure 4 in the main manuscript) and the unadjusted analysis (Figure 4).

We compare the widths of the intervals in Figure S18. In general, we observe that intervals derived from the sensitivity analysis (a covariate-unadjusted model) are wider than the original covariate-adjusted analysis. This is unsurprising, since these intervals arose from a more diffuse prior on the efficiencies. We also observed that the covariate-adjusted intervals are not uniformly narrower than the intervals obtained from fitting the unadjusted model. In fact, the

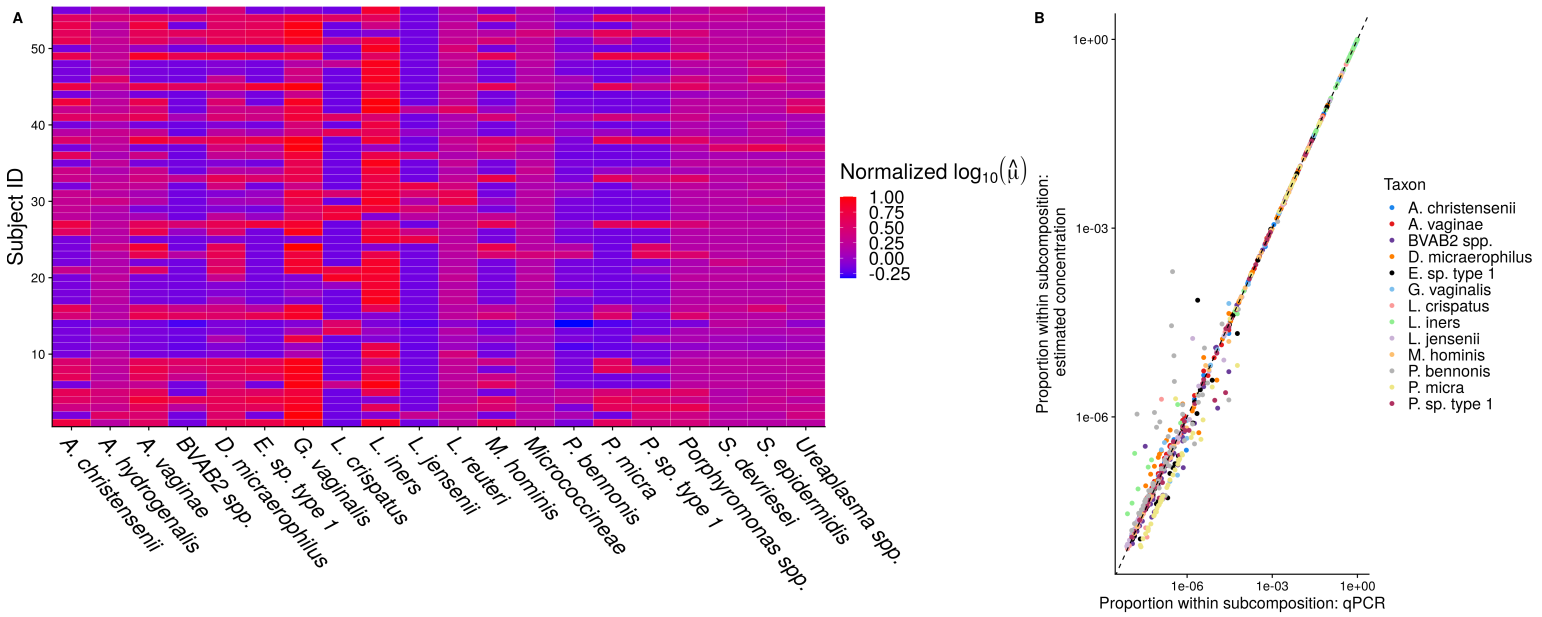

Figure S13: **A**. A heatmap showing posterior mean log concentrations for 20 taxa (the 13 taxa with observed qPCR and seven randomly-sampled taxa) and all 55 samples from the unadjusted analysis. Red indicates large concentration relative to the maximum in this subsample, while blue indicates small concentration relative to the maximum in this subsample. **B**. The relative abundance of taxa observed with qPCR versus the estimated relative abundance of the taxa based on the variable-efficiency estimator. Specifically, $V_{ij}/\sum_{k=1}^{q^{\text{obs}}} V_{ik}$ is plotted against $\hat{\mu}_{ij}/\sum_{k=1}^{q^{\text{obs}}} \hat{\mu}_{ik}$. $q^{\text{obs}} = 13$ and $n = 55$ in this dataset.

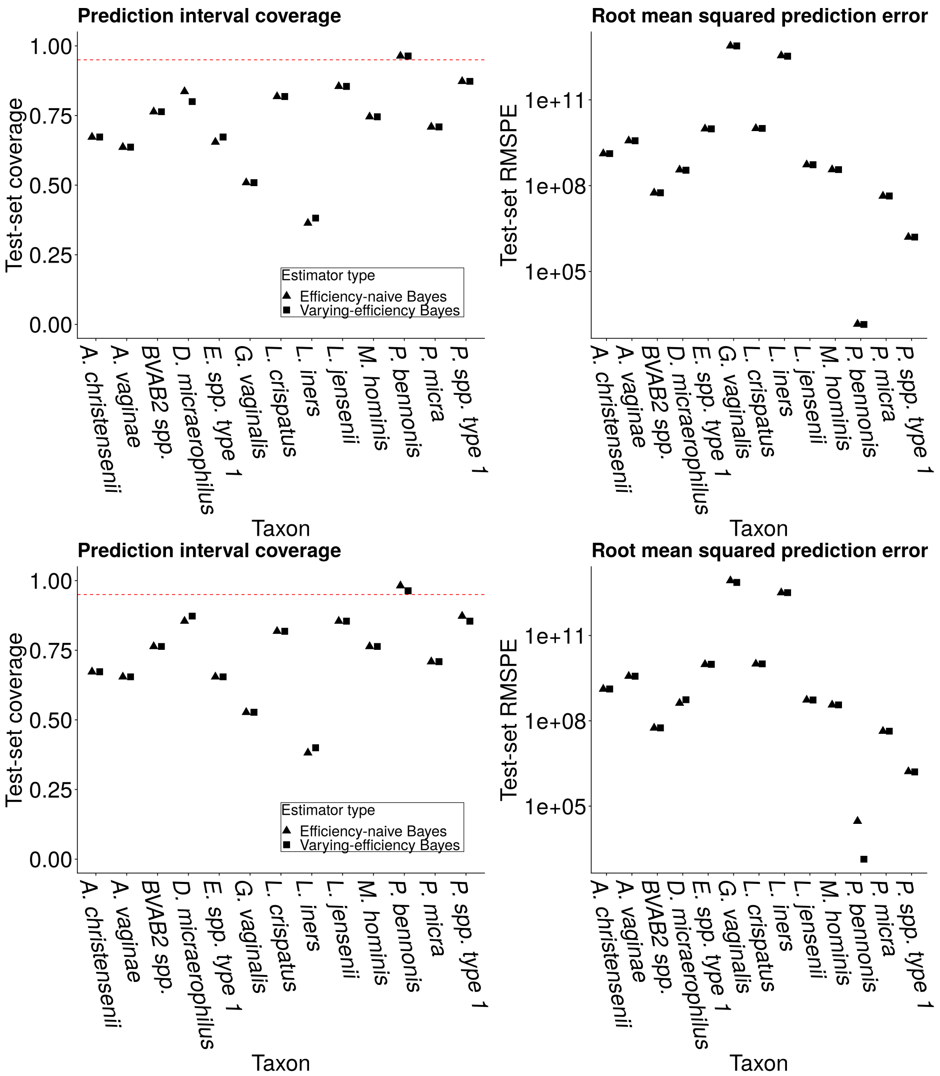

Figure S14: *Left:* Average coverage of nominal 95% prediction intervals for each taxon with observed qPCR averaged over originally withheld study participants. *Right:* MSPE for each taxon with observed qPCR. *Top row:* the unadjusted analysis. *Bottom row:* the covariate-adjusted analysis. Triangles denote the efficiency-naïve Bayesian estimator and squares denote the proposed varying-efficiency Bayesian estimator.

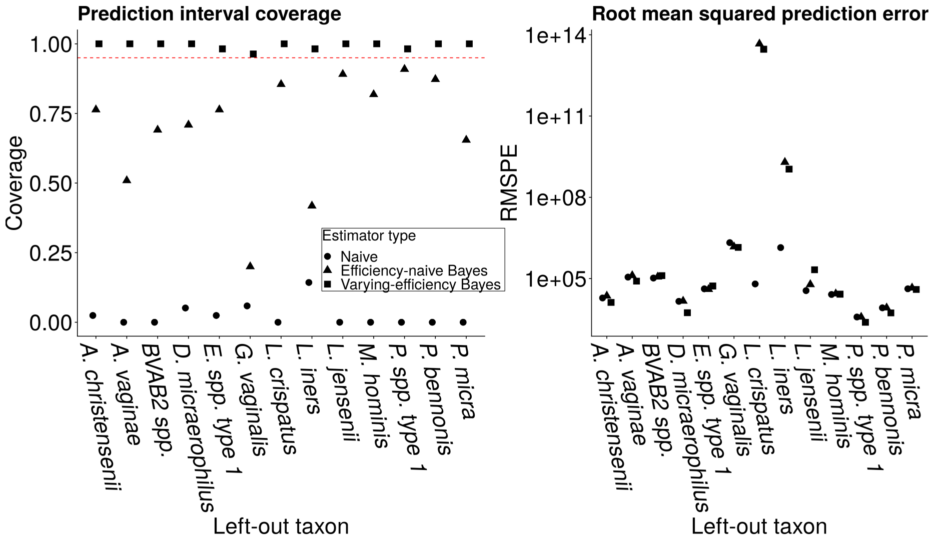

Figure S15: *Left:* Average coverage of nominal 95% prediction intervals for the left-out taxon averaged over study participants based on the unadjusted analysis. *Right:* MSPE on the left-out taxon. Circles denote the naïve estimator, triangles denote the efficiency-naïve Bayesian estimator, and squares denote the proposed varying-efficiency Bayesian estimator.

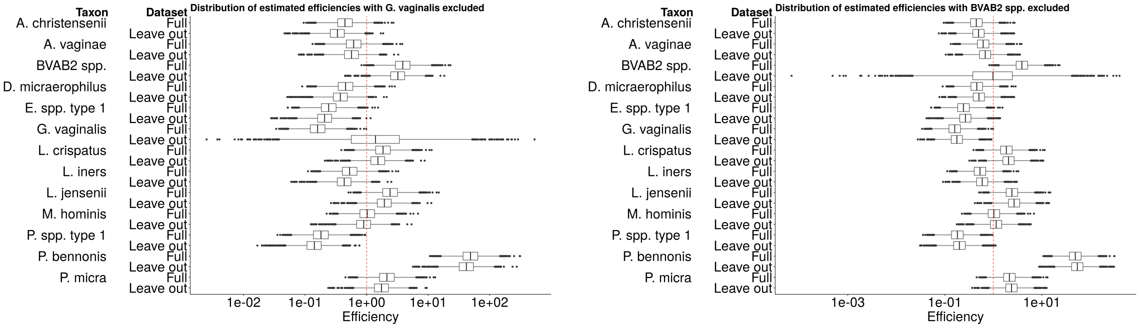

Figure S16: Boxplots showing the posterior distribution of estimated efficiencies in the unadjusted analysis. *Left:* estimated efficiencies from the full data analysis and from an analysis where *G. vaginalis* was left out. *Right:* estimated efficiencies from the full data analysis and from an analysis where *BVAB2 spp.* was left out.

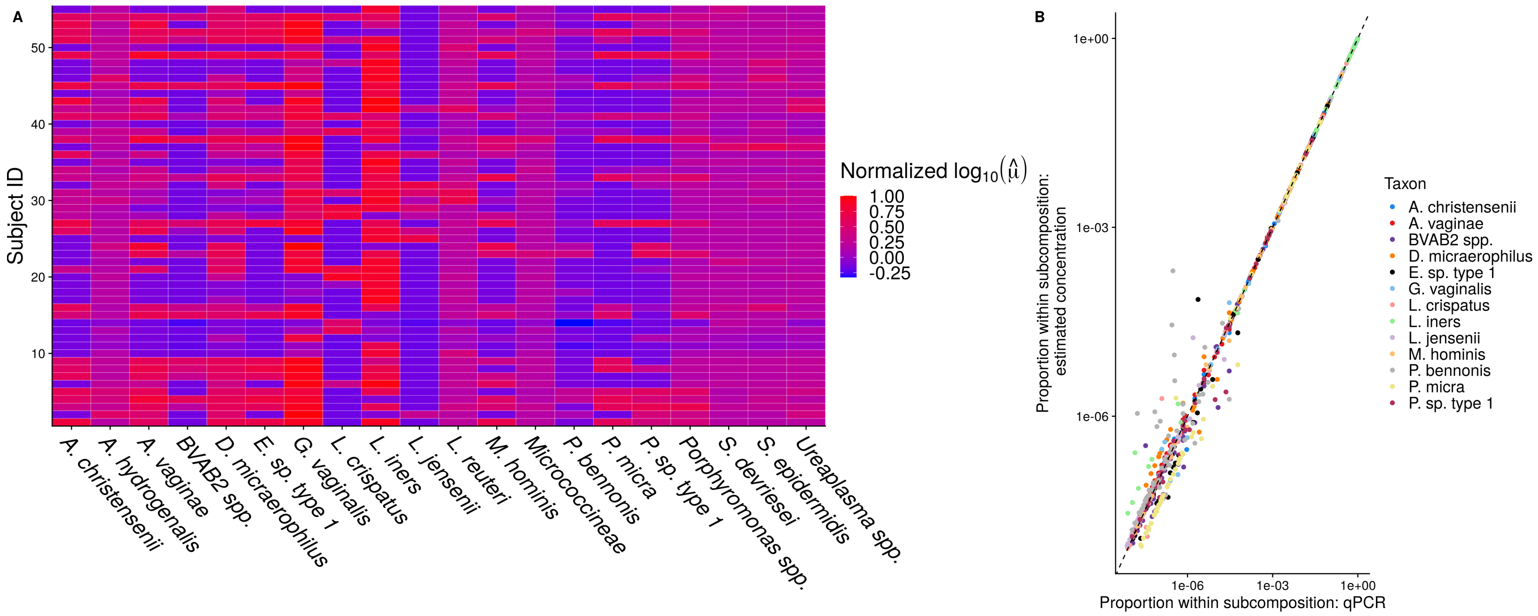

Figure S17: **A**. A heatmap showing posterior mean log concentrations for 20 taxa (the 13 taxa with observed qPCR and seven randomly-sampled taxa) and all 55 samples from the unadjusted analysis with prior parameters $\alpha_\sigma = 2$ and $\kappa_\sigma = 3$. In our original model, we chose $\alpha_\sigma = 4$ and $\kappa_\sigma = 3$ (a less diffuse prior on the e_j's). Red indicates large concentration relative to the maximum in this subsample, while blue indicates small concentration relative to the maximum in this subsample. **B**. The relative abundance of taxa observed with qPCR versus the estimated relative abundance of the taxa based on the variable-efficiency estimator. Specifically, $V_{ij}/\sum_{k=1}^{q^{\text{obs}}} V_{ik}$ is plotted against $\hat{\mu}_{ij}/\sum_{k=1}^{q^{\text{obs}}} \hat{\mu}_{ik}$. $q^{\text{obs}} = 13$ and $n = 55$ in this dataset. A color version of this figure can be found in the electronic version of the article.

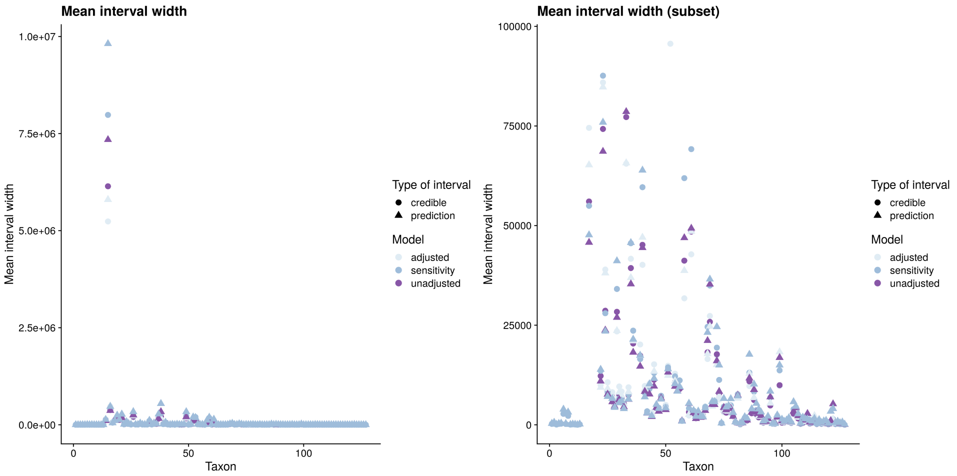

Figure S18: The widths of credible intervals for μ_{ij} (circles) and prediction intervals for V_{ij} (triangles) across 3 variations on the data analysis: the covariate-adjusted analysis described in the main text (light blue), the covariate-unadjusted analysis with varied hyperparameters described in SI Section 6 (sky blue), and the covariate-unadjusted analysis described in SI Section 6 (purple). The same data is shown with different y-axis scales in the left and right panels.

covariate-adjusted credible intervals for μ_{ij} are narrower than the unadjusted credible intervals in 61% of taxon-subject pairs (see Supplementary Data). On average the covariate-adjusted intervals were 6.4% wider than their unadjusted counterparts. This suggests that if covariate information is available, it can be used to constrain the intervals for the concentrations.

References

Ben J Callahan, Kris Sankaran, Julia A Fukuyama, Paul J McMurdie, and Susan P Holmes. Bioconductor workflow for microbiome data analysis: from raw reads to community analyses. *F1000Research*, 5:1492, June 2016.

RS McClelland, JR Lingappa, S Srinivasan, J Kinuthia, GC John-Stewart, W Jaoko, et al. Evaluation of the association between the concentrations of key vaginal bacteria and the increased risk of HIV acquisition in african women from five cohorts: a nested case-control study. *The Lancet Infectious Diseases*, 18(5):554–564, 2018.
